# Distribution of initial caries lesions in relation to fixed orthodontic therapy. A systematic review and meta-analysis

**DOI:** 10.1093/ejo/cjae008

**Published:** 2024-02-22

**Authors:** Claudia Salerno, Maria Grazia Cagetti, Silvia Cirio, Marcella Esteves-Oliveira, Richard J Wierichs, Dimitrios Kloukos, Guglielmo Campus

**Affiliations:** Department of Restorative, Preventive and Pediatric Dentistry, University of Bern, Freiburgstrasse 7, 3012 Bern, Switzerland; Department of Biomedical, Surgical and Dental Sciences, University of Milan, Via Beldiletto 1, 20142 Milan, Italy; Graduate School for Health Sciences, University of Bern, Bern, Switzerland; Department of Biomedical, Surgical and Dental Sciences, University of Milan, Via Beldiletto 1, 20142 Milan, Italy; Department of Biomedical, Surgical and Dental Sciences, University of Milan, Via Beldiletto 1, 20142 Milan, Italy; Department of Restorative, Preventive and Pediatric Dentistry, University of Bern, Freiburgstrasse 7, 3012 Bern, Switzerland; Department of Restorative, Preventive and Pediatric Dentistry, School of Dental Medicine, University of Bern, Bern, Switzerland; Department of Restorative Dentistry and Endodontology, Justus-Liebig-University Giessen, Giessen, Germany; Department of Conservative Dentistry, Periodontology and Endodontology, University Centre of Dentistry, Oral Medicine and Maxillofacial Surgery (UZMK), University of Tübingen, Tübingen, Germany; Department of Restorative, Preventive and Pediatric Dentistry, University of Bern, Freiburgstrasse 7, 3012 Bern, Switzerland; Department of Orthodontics and Dentofacial Orthopedics, School of Dental Medicine, University of Bern, Bern, Switzerland; Department of Orthodontics and Dentofacial Orthopedics, 251 Hellenic Air Force Hospital, Athens, Greece; Department of Periodontology, Faculty of Odontology, Malmö University, Malmö, Sweden; Department of Restorative, Preventive and Pediatric Dentistry, University of Bern, Freiburgstrasse 7, 3012 Bern, Switzerland; Department of Medicine, Surgery and Pharmacy, University of Sassari, 07100 Sassari, Italy; Department of Cariology, Saveetha Dental College and Hospitals, Chennai 600077, India

**Keywords:** ICLs, caries, orthodontic therapy, white spot lesion, caries incidence, caries prevalence, brackets

## Abstract

**Background:**

Initial caries lesion (ICLs) adjacent to orthodontic brackets are the most common side effect of orthodontic treatment with fixed appliances. The reported prevalence is uncertain and varies considerably across studies, from 27% to 97%.

**Objectives:**

This paper was designed to evaluate and synthesize the available evidence on the prevalence and incidence rates of ICLs in relation to orthodontic treatment. Selection criteria: The review (Prospero protocol CRD42023412952) included randomized and non-randomized clinical trials of interventions, cohort studies, and cross-sectional studies, published after 1990 on the prevalence or incidence of ICLs during or after orthodontic treatment with fixed appliances. Search methods: Pubmed, Scopus, and Embase databases were searched from 1990 until 01 May 2023. The risk of bias assessment was performed with RoB 2 and ROBINS-I tool and the Joanna Briggs Institute Critical Appraisal Checklist. Data collection and analysis: The proportion of individuals with ICLs, reported as the number/percentage of individuals/teeth with ICLs or mean number of ICLs per subject, were used to synthesize results.

**Results:**

The search yielded a total of 468 papers; 21 studies were included in the systematic review, 2 of which were not included in the meta-analysis. The prevalence rate [_95%_CI] of ICLs was 0.57% [0.48; 0.65] in 1448 patients, 0.22% [0.14; 0.33] in 11583 teeth, with a mean number of lesions equal to 2.24 [1.79; 2.70] in 484 patients evaluated. The incidence rate of new carious lesions developed during orthodontic treatment was 0.48% [0.33; 0.63] in 533 patients, 0.15% [0.08; 0.26] in 1890 teeth with a mean number of ICLs equal to 2.29 [1.12; 3.46] in 208 patients evaluated.

**Limitations:**

Although the high number of included studies and the overall good quality, there was a significant heterogeneity in the collected data.

**Conclusion:**

The prevalence and incidence rates of ICLs in subjects undergoing orthodontic treatment are quite high and raise some concerns in terms of risk assessment of orthodontic treatment. ICLs represent an alarming challenge for both patients and professionals. Effective caries prevention strategies during treatment need to be considered and implemented where appropriate.

## Introduction

Orthodontic treatment is still largely based on fixed appliances, and its duration is variable, but often quite long, reaching an average of 24 months [1]. Fixed appliances are a potential risk factor for an increased accumulation of dental plaque as the rough bracket surfaces, bands, or wires reduce natural self-cleaning and make proper brushing methods more problematic [2]. Plaque accumulation can lead to a decrease in pH, shifting the demineralization/remineralization balance towards mineral loss. If this condition persists, the development of initial carious lesions and subsequently cavitated lesions may occur [3]. The development of carious lesions around brackets and bands is a common side effect of fixed orthodontic treatment [4]. Early non-cavitated carious lesions are also referred to as white spot lesions (WSLs) because of their characteristic chalky white appearance [5, 6]. As the term WSLs refers only to the colour of the lesion and can be confused with other types of dental defects, such as dental fluorosis or MIH, it has recently been proposed to replace it with the term Initial Caries Lesions (ICLs) [7]. The white appearance can be physically explained by an increased light scattering due to air and saliva inclusions within the lesion body [8]. ICLs are regarded as a public health problem as they are the first sign of carious lesions and, as mentioned, may evolve into cavitated lesions if left untreated. However, even if they do not progress to cavitated carious lesions, ICLs can still pose an aesthetic problem, especially if they are located in the anterior teeth [9]. Such lesions may become evident as early as one month after the placement of fixed appliances [10]. ICLs are generally found on the vestibular surfaces of the teeth, around the brackets, particularly in the gingival area, with a higher prevalence on the lateral incisors of the maxillary arch and the premolars of the mandibular arch [11, 12]. Despite several studies having described the development model of ICLs, this adverse event remains an unresolved problem [13, 14]. The reported prevalence of ICLs associated with orthodontic treatment varies widely in the literature, and this variability can be attributed to differences in the type of teeth examined, the modalities of examination and scoring, the different demographic characteristics of the patients (age, ethnicity, socioeconomic status), the duration of treatment, and the characteristics and materials used in the orthodontic appliance [15, 16]. The first clinical-visual method for assessing ICLs, the Gorelick Index, was developed in 1982 [4]; later, the index was modified including the extension of the lesion in the assessment [17]. In addition to clinical methods, the use of imaging software has been proposed to assess the presence and severity of ICLs in digital photographs has been proposed. Quantitative light-induced fluorescence, intraoral scanners, and fluorescence-based devices have also been used as alternatives to clinical assessment [18–20]. These different assessment methods have increased the variability found in the reported prevalence and incidence of ICLs.

To date, only one systematic review with meta-analysis has been published to describe the prevalence and incidence of white spot lesions in subjects undergoing fixed orthodontic treatment [21]. However, the data date back to 2015, and the study also included retrospective papers and papers in which fluoride was administered, which due to its preventive action could have led to bias in the results.

Based on these premises, the purpose of this systematic review was to evaluate, synthetize and analyse the available evidence, updating data on the prevalence and/or incidence rates of ICLs in relation to orthodontic treatment with fixed appliances.

## Materials and methods

### Protocol registration

The present systematic review was registered a priori in the International Prospective Register of Systematic Reviews (PROSPERO N= CRD42023412952; https://www.crd.york.ac.uk/prospero/display_record.php?RecordID=412952), and it has been conducted and reported according to the Cochrane Handbook of Systematic Reviews of Interventions and to the guidelines of Preferred Reporting Items for Systematic Review and Meta-Analysis (PRISMA). The PRISMA checklist is displayed in Supplementary Table S1.

### PECO question

The research question was formulated following the PECO model as follow: ‘what are the incidence and prevalence of initial caries lesions during or following orthodontic treatment with fixed appliances?’:

- **P**articipants/population: subjects of any age undergoing/underwent fixed orthodontic treatment on permanent dentition;
- **E**xposure: orthodontic treatment with fixed orthodontic appliances;
- **C**omparator/control: no intervention or not applicable;
- Primary **O**utcomes: prevalence and/or incidence of ICLs at subject level and at teeth surface level;
- Secondary **O**utcomes: influence of demographic or treatment-related factors on prevalence and incidence of ICLs.
- **S**tudy design: Placebo group of randomized (RCT) and non-randomized clinical studies (NRSI), cohort studies and cross-sectional studies.

### Eligibility criteria

Placebo group of randomized (RCT) and non-randomized clinical studies (NRSI), cohort studies, and cross-sectional studies, published in English language after 1990 on prevalence or incidence of initial caries lesions during or after orthodontic treatment with fixed orthodontic appliances during permanent dentition were the inclusion criteria of the present review.

The following exclusion criteria were applied:

- Studies for which full text was not available.
- Studies not in English language
- Type/design of study: *in vitro* studies, case report or case series, retrospective studies, studies with a split-mouth design.
- Type of orthodontic appliance: self-ligating brackets, lingual fixed orthodontic appliances, transparent aligners.
- Studies in which the pre- and post- treatment assessment was carried without using the same assessment method.
- Studies in which fluoridated products were provided during orthodontic treatment in addition to fluoridated toothpaste.
- Studies with orthodontic treatment duration of less than 6 months.
- Studies in which no outcome information was reported.

### Information sources, search strategy, and selection process

Three electronic databases, PubMed, Embase, and Scopus were searched from 1990 until 01 May 2023 by two authors (C.S. and S.C.). The search strategy included a search string for each database:

- For PubMed, the string used was: ((prevalence[Title/Abstract]) OR (incidence[Title/Abstract])) AND ((white spot[Title/Abstract]) OR (caries, dental[MeSH Terms]) OR (enamel lesio*[Title/Abstract]) OR (uncavitated[Title/Abstract]) OR (initial caries[Title/Abstract])) AND ((orthodontic[Title/Abstract]) OR (aligners[Title/Abstract]) OR (orthodontic bracket[MeSH Terms]) OR (orthodontic brackets[MeSH Terms]) OR (bracket, orthodontic[MeSH Terms]) OR (brackets, orthodontic[MeSH Terms]) OR (lingual orth*[Title/Abstract]) OR (vestibular orth*[Title/Abstract]))
- For Embase: (‘prevalence’/exp OR prevalence OR incidence) AND (‘white spot lesion’ OR ‘initial dental caries’ OR ‘enamel lesion’ OR ‘non cavitaded’ OR uncavitated OR ‘initial caries’ OR ‘white spot’) AND (orthodontics OR aligners OR ‘orthodontic bracket’)
- For Scopus: (TITLE-ABS-KEY (prevalence) OR TITLE-ABS-KEY (incidence)) AND (TITLE-ABS-KEY (‘white spot’) OR TITLE-ABS-KEY(‘initial caries’) OR TITLE-ABS-KEY(‘enamel lesion’) OR TITLE-ABS-KEY(uncavitated)) AND (TITLE-ABS-KEY(‘Orthodontic treatment’) OR TITLE-ABS-KEY(‘Orthodontic brackets’) OR TITLE-ABS-KEY(‘lingual orthodontic’) OR TITLE-ABS-KEY(‘vestibular orthodontic’) OR TITLE-ABS-KEY(aligners) OR TITLE-ABS-KEY(brackets)).

The search strategy was initially developed for PubMed using keywords and MeSH terms and adapted to the other databases. Cross-referencing was also performed using the reference lists of full-text papers, and grey literature was retrieved via opengrey.eu (http://www.opengrey.eu). Studies were managed on Rayyan reference management software [22], where duplicate studies were removed.

At all phases, the reviewers were trained, and a pilot test was conducted to audit the eligibility criteria. In phase one (title and abstract reading) and phase two (full-text reading), the studies were assessed for eligibility by two independent reviewers (CS and MGC). Disagreements were solved in a consensus meeting, and if any disagreement persisted, another reviewer (SC) was involved to steer the decision. After screening four pilot studies, four reviewers (SC, DK, MEO, and RJW) independently collected data in a self-designed Excel spreadsheet (Microsoft Excel®) (Supplementary Table S2).

### Data items and effect measures

The following data were obtained: authorship, year and country of publication, journal, study design, the main purpose of the study, sampling strategy, and characteristics of the sample (size, gender, age, type, and number of teeth included), blinding, follow-up, evaluation of ICLs at baseline, type of evaluation, type of assessment (index), type of orthodontic appliance, treatment duration, baseline clinical evaluation, fluoride supplement administration, water fluoridation, results of primary and secondary outcome of each study. Primary outcomes were prevalence and/or incidence of ICLs at subject and/or tooth surface level;

Secondary outcomes were the influence of demographic or treatment variables on the prevalence and incidence of ICLs. Data of prevalence and incidence of ICLs as percentage per subject, per surface, and mean number per subject were extracted and rounded up to two decimals; if this was not possible, data were extracted as they were reported in the papers. If the averages or percentages of the outcomes considered were not clearly presented in the article they were computed from the raw data where present. When necessary, for absent or incomplete data, the correspondence author was contacted via email. Two attempts were made to contact him/her. In the absence of answers, the data were not included.

### Risk of bias assessment

The risk of bias assessment was carried out by three reviewers independently (MEO, SC and MGC), using the Cochrane collaboration’s RoB 2 and ROBINS-1 tool for RCT and NRSI studies, respectively. The Excel tool for RoB 2 was used to input answers given to signalling questions, and then an algorithm estimated the overall risk of the bias according to the results for each domain as: ‘low risk’, ‘some concerns’ or ‘high risk’. Risk of bias plots were drawn using the Cochrane robvis web app [23]. The ROBINS-I tool was used to assess the risk of bias for non-randomized studies of intervention (NRSI) [24]. The authors answered to signalling questions in each domain and then estimated the overall risk of bias as: ‘low’, ‘moderate’, ‘serious’, or ‘critical’. The Joanna Briggs Institute Critical Appraisal Checklist for Studies Reporting Prevalence Data [25] was applied to assess the methodological quality of the cross-sectional and longitudinal studies without control group. This tool comprises nine questions that assess the methodological quality of studies considering sample characteristics, sampling method, sample size, participants’ description, statistical analysis, validity, reliability of condition under study, and response rates. All questions can be answered as ‘yes’, ‘no’, ‘unclear’ or ‘not applicable’. Two reviewers (S.C. and M.G.C.) were previously trained and calibrated to use this tool, discussing each predetermined question. In case of disagreements, a third reviewer (S.C.) was involved to steer the decision.

The Grading of Recommendation, Assessment, Development, and Evaluation (GRADE) tool was used to assess the certainty of the evidence, using the GRADE-PRO website (https://www.gradepro.org/, accessed on 14 September 2023). In the absence of a formal procedure for the assessment of certainty in prevalence estimates, the framework developed for the incidence estimates was applied in the context of prognostic studies. The greatest evidence for a prevalence meta-analysis comes from cross-sectional studies or baseline examinations in cohort studies. Thus, the assessment of evidence from these types of studies begins with a ‘high certainty of evidence’, and is downgraded depending on the risk of bias, inconsistency, indirectness, imprecision, and publication bias. Finally, the level of certainty among the selected aspects of the evidence can be classified as high, moderate, low, or extremely low. The assessment was performed independently by two authors (G.C., M.E.O.). In cases of disagreement, a third review author was involved (D.K.).

### Synthesis of results

Only studies with an RCT, NRSI, cross-sectional or longitudinal design, reporting the final sample size, mean or median age, and the proportion of individuals with ICLs, reported as the number/percentage of individuals/teeth with initial dental caries or mean number of ICLs per subject, were included in the meta-analysis. Prometa3 Software® (Internovi, 2015) was used for the meta-analysis that was performed if three or more studies included comparable findings. The sample size together with the number of subjects and/or teeth with ICLs, mean number of teeth affected per subject were extracted or calculated for each study and for each outcome variable to be meta-analysed. Statistical heterogeneity of effects among studies was assessed by means of the Cochran’s test, with a significance threshold of *P* < .05. The percentage of variability in the effect estimates due to heterogeneity rather than chance was calculated with *I*^2^ statistic. Due to high clinical and methodological heterogeneity, meta-analysis was undertaken using a random effects model. The results of each meta-analysis were graphically presented by Effect Size of Forest plots. The collected independent variables such as age and duration of treatment were associated with the primary outcomes performing sub-group metanalyses. The level of oral hygiene could not be associated due to a lack of sufficient data.

Publication bias was evaluated using a funnel plot approach, and Begg’s and Egger’s correlation test were performed to identify asymmetry. If any asymmetry was identified, the included studies were checked, assessing whether the asymmetry was due to publication bias or other reasons, such as the presence of methodological heterogeneity. When the meta-analysis appeared inappropriate, the results of the included studies were not pooled, and a qualitative description with supporting data was presented. A *P*-value of .05 or less was considered statistically significant for all analyses.

## Results

### Study selection

**Figure 1.**
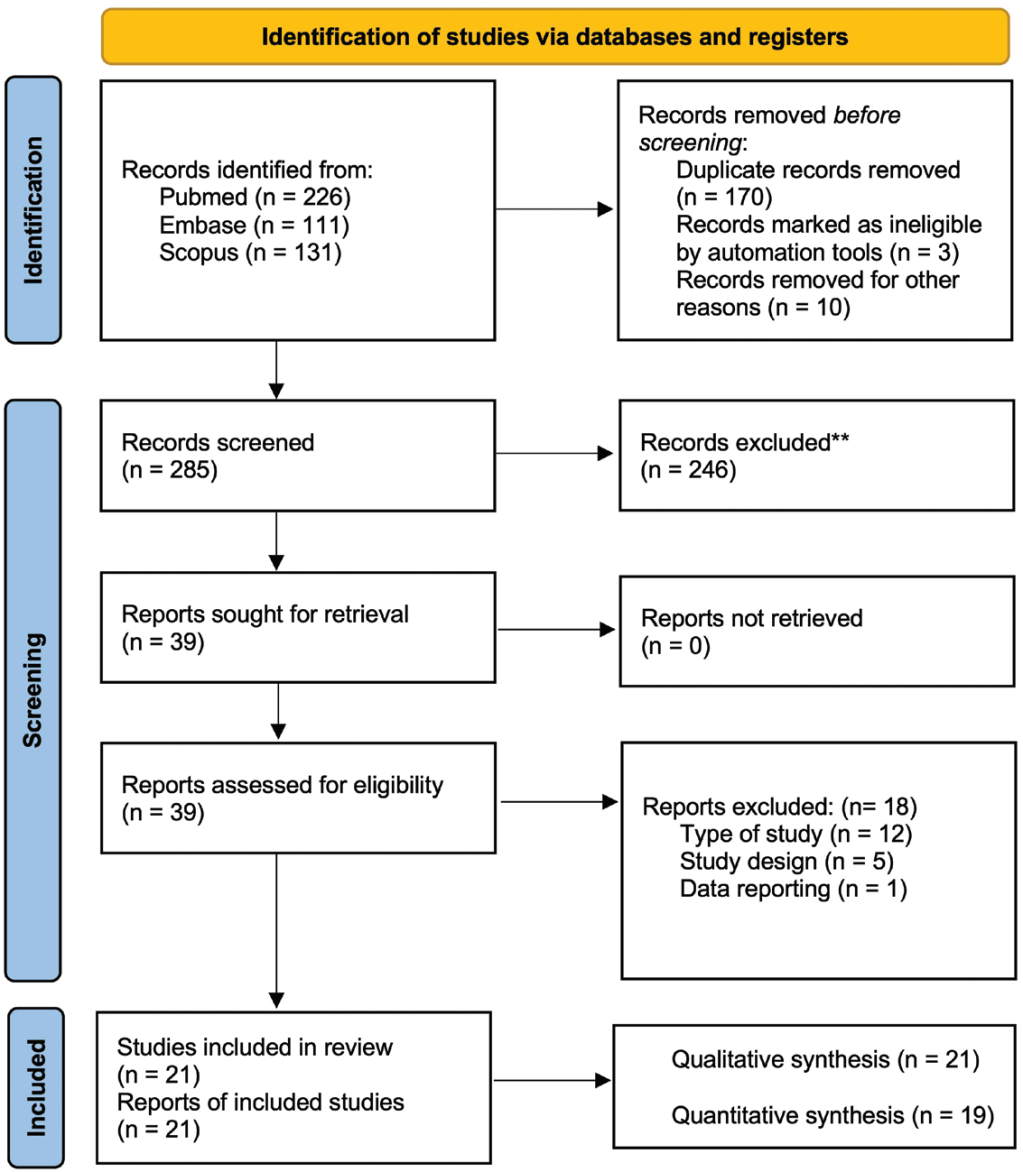
PRISMA 2020 flowdiagram for new systematic reviews.

The initial literature search yielded a total of 468 papers; after eliminating duplicates and ineligible studies by title or abstract 285 papers were evaluated; 246 studies were excluded with a proportional agreement between reviewers of 93.00% and a Cohen’s *K* of 0.82. Therefore, 39 studies proceeded to full-text assessment, and after full-text evaluation, 21 were included in the present systematic review, 19 of which were included in the meta-analysis (Fig. 1). The proportionate agreement at this stage between reviewers was 94.91% with a Cohen’s factor of 0.88. The list of excluded papers and the reason for exclusion can be found in Supplementary Tables S3 and S4).

### Studies and sample characteristics

**Figure 2.**
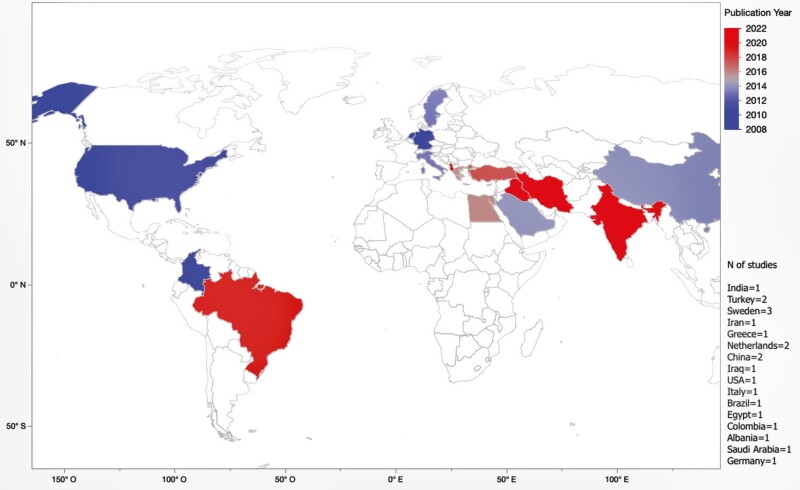
Geographic location and year of publication of included studies.

The selected studies were conducted in Albania [26], Brazil [27], China [28, 29], Colombia [30], Egypt [31], Germany [32], Greece [33], India [34], Iran [35], Iraq [36], Italy [12], Netherlands [37, 38], Saudi Arabia [39], Sweden [40–42], Turkey [43, 44], and USA [45] (Fig. 2). Papers were published between 2005 and 2022: 16 studies were published in the last 10 years [12, 26–29, 31, 33–36, 38, 39, 41–44] and 5 studies were published before 2012 [30, 32, 37, 40, 45] (Table 1). Of the included studies, 11 were RCTs [28, 31, 33–35, 38, 40–44], of which only 1 (41) was a multicenter study; 7 studies were NRSIs [12, 27, 30, 32, 36, 39, 45] and finally, 3 were observational studies with no control group [26, 29, 37].

**Table 1.** General characteristics of the studies included.

Authors (year)	Journal	Study location	Type of study	Blinding	Treatment duration before ICLs assessments
Toti *et al*. (2022)	Healthcare	Albania	Observational study with no control group	No	3 m; 6 m
Pinto *et al*. (2018)	Caries Research	Brazil	Nonrandomized study of Intervention	No	12 m; 24 m; 36 m
Jiang *et al*. (2013)	Pediatric Dentistry	China	Randomized Controlled Trial	Double	≥12 m
Jiang *et al*. (2015)	The Chinese Journal of Dental Research	China	Observational study with no control group	No	≥12 m
Martignon *et al*. (2010)	Community Dental Health	Colombia	Nonrandomized study of Intervention	No	≥12 m
Hammad *et al*. (2016)	Journal of Orofacial Orthopedics	Egypt	Randomized Controlled Trial	Single	≥12 m
Heinig and Hartmann (2008)	Journal of Orofacial Orthopedics	Germany	Nonrandomized study of Intervention	No	≥18 m
Gizani *et al*. (2016)	European Journal of Orthodontics	Greece	Randomized Controlled Trial	Double	≥12 m
Ravikiran *et al*. (2021)	International Journal of Dentistry and Oral Science	India	Randomized Controlled Trial	Double	≥12 m
Najafi *et al*. (2022)	European Journal of Orthodontics	Iran	Randomized Controlled Trial	Triple	3 m; 6 m; ≥ 12 m
Mohammed *et al*. (2021)	International Medical Journal	Iraq	Nonrandomized study of Intervention	No	≥12 m
Lucchese *et al*. (2013)	European Journal of Orthodontics	Italy	Nonrandomized study of Intervention	No	6 m; 12 m
Boersma *et al*. (2005)	Caries Research	Netherlands	Observational study with no control group	No	≥18 m
Van der Kaaij *et al*. (2015)	European Journal of Oral Sciences	Netherlands	Randomized Controlled Trial	Double	≥18 m
Almosa *et al*. (2014)	Angle Orthodontis	Saudi Arabia	Nonrandomized study of Intervention	No	≥18 m
Stecksén-Blicks *et al*. (2007)	Caries Research	Sweden	Randomized Controlled Trial	Double	≥6 m
Sonesson *et al*. (2014)	European Journal of Orthodontics	Sweden	Multicenter Randomized Controlled Trial	Single	≥18 m
Sonesson *et al*. (2020)	European Journal of Orthodontics	Sweden	Randomized Controlled Trial	Triple	≥18 m
Esenlik *et al*. (2016)	European Journal of Paediatric Dentistry	Turkey	Randomized Controlled Trial	Not specified	≥18 m
Mahmoudzadeh *et al*. (2019)	Turkish Journal of Orthodontics	Turkey	Randomized Controlled Trial	Double	6 m
Tufecki *et al*. (2011)	Angle Orthodontist	USA	Nonrandomized study of Intervention	Single	6 m; 12 m

In order to include large sample controlled high-quality studies, randomized controlled trials whose primary outcome was to assess the efficacy of remineralizing products on the incidence of ICLs were also considered, including only the untreated placebo group. In the intervention groups, different types of products/device were applied including CPP-ACP [43], fluoride mouthwash [34, 38], fluoride varnish [40, 42], Laser Co_2_ [44], high fluoride toothpaste 5000 ppm [41], Xylitol and fluoride varnish [35], probiotic intake [33], fluoride foam [28], and sealant application [31] (Table 2, Supplementary Table S5).

**Table 2.** Main characteristics and summary of findings of the included studies.

Author (Year)	*N* subject evaluated (total study sample)	N teeth evaluated (total study sample)	Study design	ICLs index	Prevalence/Incidence	Primary outcome	Results of primary outcome
Toti *et al*. (2022)	74	1776	Before-after treatment	ICDAS	Prevalence	ICLs prevalence and distribution	Prevalence 60.8%; surface prevalence 9.96%; mean 2.39 ± 2.97
Pinto *et al*. (2018)	195 (260)	n.a.	No treatment vs 1year vs 2 years vs, 3 years	Maltz	Prevalence	ICLs prevalence	Prevalence 1 year: 27.7%; mean 0.61I _95_CI [0.44;0.84] 2 years: 72.3%; mean 2.14 _95_CI[1.80;2.53] 3 years: 72.3; mean 1.95 _95_CI[1.63;2.32]
Jiang *et al*. (2013)	47 (95)	845 (1685)	Placebo vs fluoride foam	Gorelick	Both	ICLs prevalence and incidence	Prevalence 64%; mean 4.79 ± 5.58—incidence 51%; mean 4.36 ± 5.41
Jiang *et al*. (2015)	202	5612	ICLs evaluation at debonding	Gorelick	Prevalence	ICLs prevalence	Prevalence 57.9%; surface prevalence 17.3%; mean 4.8
Martignon *et al*. (2010)	74 (137)	n.a.	Treatment vs no treatment	ICDAS	Prevalence	ICLs prevalence	Prevalence 96%; mean 11.3 ± 7.1;
Hammad and Knösel (2016)^*^	21 (42)	n.a.	Control vs sealant	Non-standardized	Incidence	ICLs incidence	n.a.
Heinig and Hartmann (2008)	40 (78)	800 (5788)	Control vs sealant	Combined index system	Incidence	ICLs incidence	Incidence 85.71%; surface incidence 9.18%
Gizani *et al*. (2016)	43 (85)	n.a.	Placebo vs probiotic *Lactobacillus reuteri*	Gorelick	Incidence	ICLs incidence	Incidence 39.53%; mean 1.7 ± 2.5
Ravikiran *et al*. (2021)	25 (50)	250 (500)	Control vs fluoride rinse	ad hoc software	Both	Efficacy of amine fluoride mouthwash on ICLs reduction	Prevalence 2.46 ± 1.87 – incidence 0.55 ± 0.43
Najafi *et al*. (2022)	29 (115)	290 (1200)	Placebo vs 10% xylitol vs 20% xylitol vs 5% fluoride varnish	LF pen and Gorelick	Incidence	ICLs incidence with LF pen and visual evaluation	Surface incidence 31.4% - LF value mean 4.30 ± 1.59
Mohammed *et al* (2021)	120 (170)	n.a.	No treatment vs 6 mo treatment, vs 12 mo treatment	Gorelick	Prevalence	ICLs prevalence	Prevalence 6 mo 38,33% prevalence 12 mo 46.66%
Lucchese and Gherlone (2013)	123 (191)	n.a.	No treatment vs 6 mo treatment, vs 12 mo treatment	Gorelick	Prevalence	ICLs prevalence	Prevalence 6 mo 40,67% prevalence 12 mo 43,75%
Boersma *et al*. (2005)	62	1536	Visual evaluation vs QLF evaluation	Yes/no	Prevalence	QLF VS visual examination	Prevalence 97%; surface prevalence 18,48% - higher number of ICLs with QLF examination
van der Kaaij *et al*. (2015)^*^	45 (81)	n.a.	Placebo vs fluoride rinse	ICDAS	Incidence	ICLs incidence	Mean ∆ F = 10.3%± 3.0%.
Almosa *et al*. (2014)	89	G 822 P 831	governmental (G) patients vs private (P) patients	ICDAS and LF pen	Prevalence	ICLs prevalence with LF pen and visual evaluation	G group prevalence 91.1%; surface prevalence 50.2% P group prevalence 56.8%%; surface prevalence 15.3%
Stecksén-Blicks *et al*. (2007)	125 (257)	n.a.	Placebo vs: fluoride varnish	Gorelick	Both	ICLs prevalence and incidence	Prevalence 29.7% - incidence 25,7%
Sonesson *et al*. (2014)	192 (380)	n.a.	Control vs high-fluoride toothpaste	Gorelick	Both	ICLs prevalence and incidence	Prevalence 45,3%; mean 1.2 ± 1.8 - incidence 26,6%
Sonesson *et al*. (2020)	73 (148)	730 (1480)	Placebo vs fluoride varnish	Gorelick	Both	ICLs prevalence and incidence	Prevalence 43.83%; surface prevalence 43,56% - incidence 37.87%; mean 1.9 ± 2.5
Esenlik *et al*. (2016)	20 (40)	542 (1088)	Control vs CPP-ACP paste	Gorelick	Incidence	ICLs incidence	Incidence 80.00%; surface incidence 15.31%; mean 4.1 ± 4.0
Mahmoudzadeh *et al*. (2019)	47 (95)	276 (554)	Control vs laser Co2	ad hoc software and enamel decalcification	both	ICLs prevalence and incidence	Surface prevalence 15.2% - surface incidence 8.7%
Tufekci *et al*. (2011)	72 (100)	n.a.	No treatment vs 6 mo treatment, vs 12 mo treatment	Non-standardized	Prevalence	ICLs prevalence	Prevalence 6 mo 37.83%; mean 0.92 ± 0.22 prevalence 12 mo 45.71%; mean 1.13 ± 0.22

^*^Studies excluded from metanalysis: M±SD=mean ± standard deviation; N=number; ICLs=Initial Caries Lesions; n.a.= not available; _95_CI = 95% Confidence Interval; ICDAS= International Caries Detection System.

Among NRSIs, one study compared public health and private orthodontic patients to investigate whether the setting of care played a role in the development of ICLs [39], one study aimed to assess the efficacy of sealant application [32] and five studies compared subjects under orthodontic treatment and subjects not under treatment [12, 27, 30, 36, 45].

The included sample sizes ranged from 20 [43] to 202 [29] subjects, with seven studies including only adolescents [12, 31, 38, 40–43]. In all studies, samples included both sexes.

Baseline evaluation included caries experience, assessments using DMFT/DMFS and/or ICDAS [26, 28, 30, 38, 40, 43], and periodontal health and/or oral hygiene status [26, 27, 29–31, 33, 37, 38, 43].

### Initial caries lesions evaluation

The method used for the ICLs detection was either by inspection/visual/clinical only in 11 studies [12, 26–30, 35, 36, 39, 41, 45] and by dental photographs only in 6 studies [31, 34, 40, 42–44]. A combination of clinical and photographic evaluation was used for ICLs detection in two studies [32, 33]. Clinical detection and quantitative light-induced fluorescence were used in two studies [37, 38]. Drying before evaluation was done in almost all studies, except for four studies in which it was not specified [31, 34, 37, 43].

A substantial heterogeneity was found in the index used for the lesions assessment: Gorelick index was used in 10 studies [12, 28, 29, 33, 35, 36, 40–43], ICDAS index was used in 4 studies [26, 30, 38, 39]; Maltz index was used in one study [27]; Laser Fluorescence pen was used additionally in 2 studies [35, 39]; a combined index system (Årtun & Brobakken + Banks & Richmond + Zachrisson & Zachrisson + Gorelick) was used in one study [32]; two studies used an ad hoc software [34, 44]; a yes/no evaluation was used in one study [37] and, finally a non-standardized index was used in 2 studies [31, 45].

### Prevalence and incidence of ICLs

The prevalence of ICLs reported ranged from 27.70% [27] to 97.00% [37], while the reported incidence ranged from 26.6% [41] to 85.7% [32].

The contribution of age was not clear: a higher prevalence of lesions was reported among participants in the 11–15 age range (62.8%), when compared with the 16–24 range (47.7%) [29]. Moreover, a higher prevalence has been described among participants over 20 years of age as the length of orthodontic treatment may increase [27]. No significant differences were found between males and females in ICLs distribution in four studies [13, 26, 29, 36], even if a higher percentage of lesions was found in males in three studies [13, 37, 45].

A significantly higher prevalence of ICLs was reported in patients treated in public health centres than in those treated in private centres [39]. Only few studies investigated the oral hygiene status as a risk factor for the development of ICLs [26, 27, 29–31, 37] and a higher frequency of daily tooth brushing and/or good oral hygiene index were associated to a lower number of ICLs. Upper lateral incisors resulted in the teeth more affected by ICLs, followed by canines and premolars in few studies [29, 40–43]; other studies described a higher percentage of ICLs in the mandibular first molars [30] or in the maxillary first molar [26]. ICLs were mostly located in the middle third of the surface [30] or in the gingival region [32]. One study did not observe statistically significant differences in the distribution of ICLs among different types of teeth [45].

Several studies have examined the use of fluoride as a preventive measure during orthodontic therapy: professional application of a fluoride varnish every 2–3 months significantly reduces the development of the lesions [35, 40, 42]; fluoride rinses were capable to obtain a slight reduction in the incidence of ICLs [34, 38]; a substantial reduction of the lesions was observed with fluoride foam applied every 2 months professionally [28]; high fluoridate toothpaste [41] or a fluoride-releasing bond applied to the entire buccal surface as a sealant was also successful to reduce the incidence of ICLs [32].

### Quality assessment of included studies

**Figure 3.**
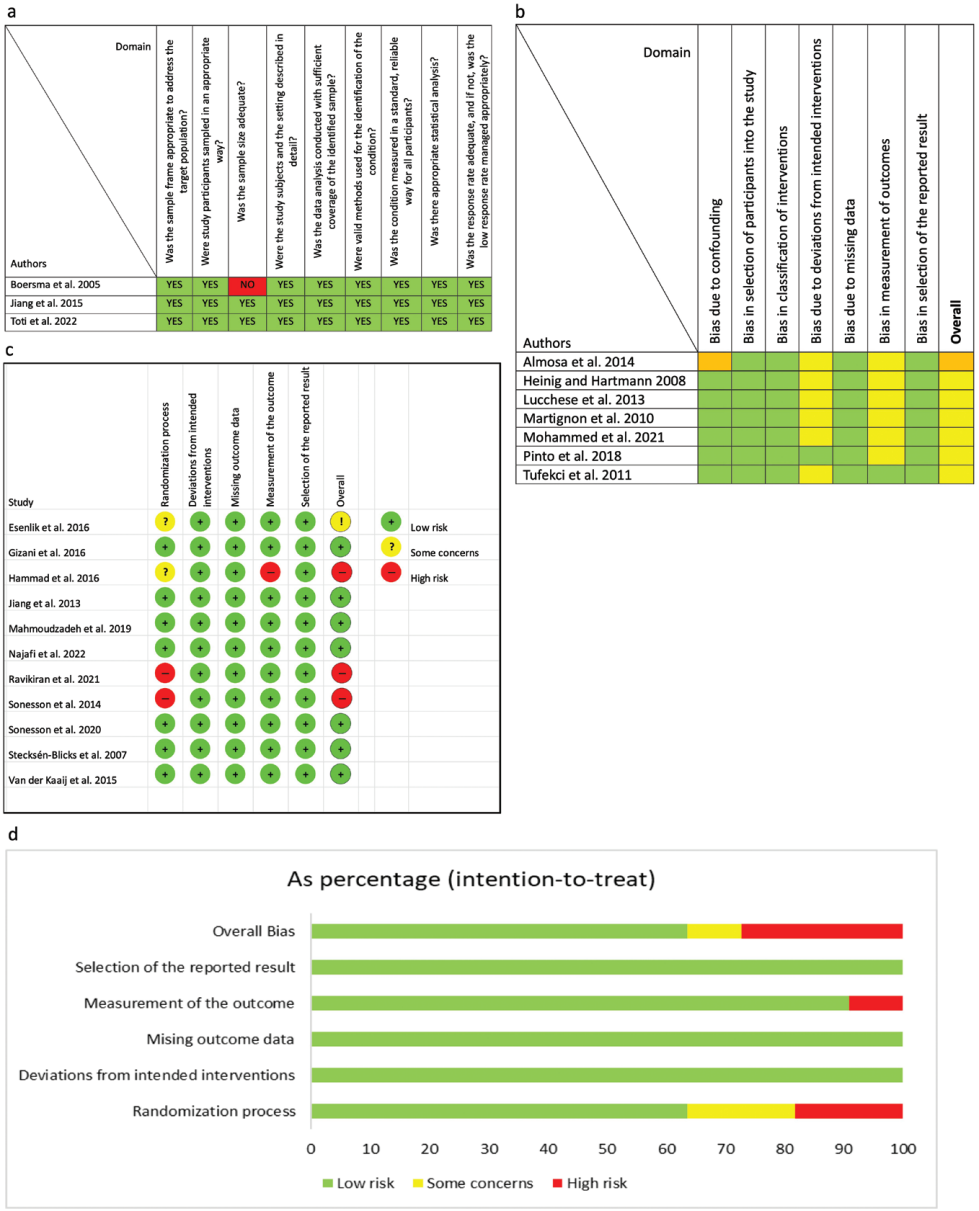
(a) Quality assessment of before-after studies with no control group, observational cohort studies; (b) Risk of bias with ROBINS-1 tool of NRSIs studies; (c) Risk of bias with Rob2 tool of RCTs studies.

The quality assessment of before-after studies with no control group, observational cohort studies, and cross-sectional studies is shown in Fig. 3a. Two studies [26, 29] were judged to be of high quality and one [37] study showed some concerns due to inadequate sample size. Of the seven NRSIs included (Figure 3b), six [12, 27, 30, 32, 36, 45] have moderate and one [39] severe risk of bias, due to confounding and/or to deviations from intended interventions and in selection of the reported result. Of the 11 RCTs (Figure 3c and 3d), seven [28, 33, 35, 38, 40, 42, 44] were judged to have a low risk of bias; one [43] showed same concerns due to randomization process and three [31, 34, 41] showed a high risk of bias due to randomization process and/or measurement of the outcomes.

The GRADE approach was used to assess 14 outcomes. All analyses were categorized as low or very low level of certainty, which means the true effect may be substantially different from the estimate of the effect. GRADE Summary of Findings Table for the Outcomes of the Systematic Review and Meta-Analysis was reported in (Supplementary Tables S6 and S7).

### Meta-analysis

**Figure 4.**
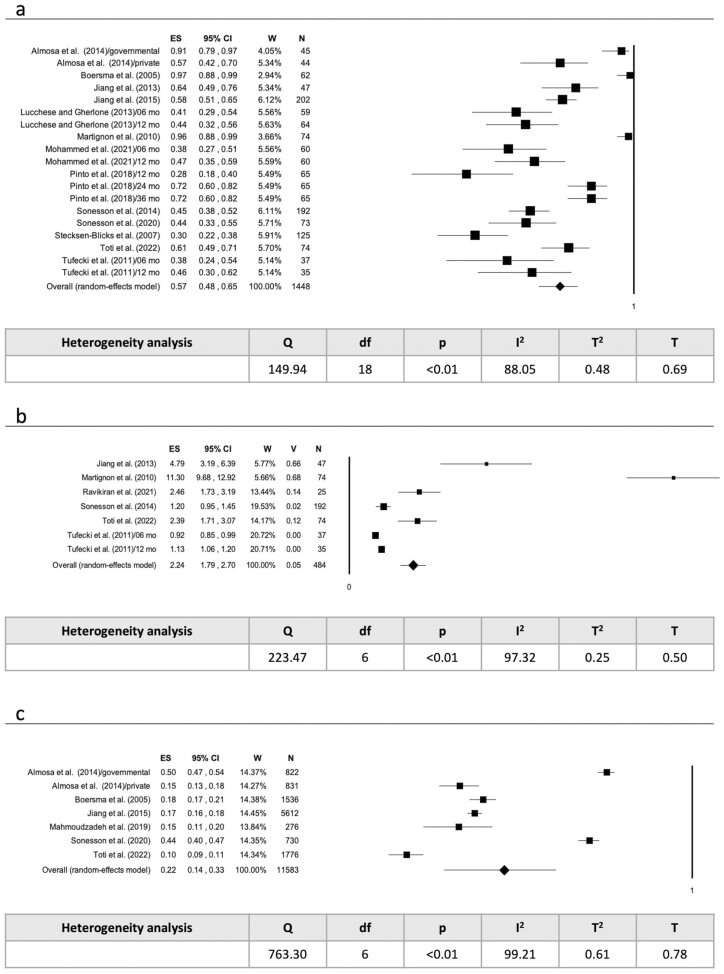
(a) Pooled prevalence as percentage per subjects; (b) pooled prevalence as mean per subject; (c) pooled prevalence as percentage per surface. Legend: ES = Effect Size; CI = Confidence Interval; W = Weight; V = Variance; N = subject/surface; Sig = significance; df = degree of freedom; T = Tau.

**Figure 5.**
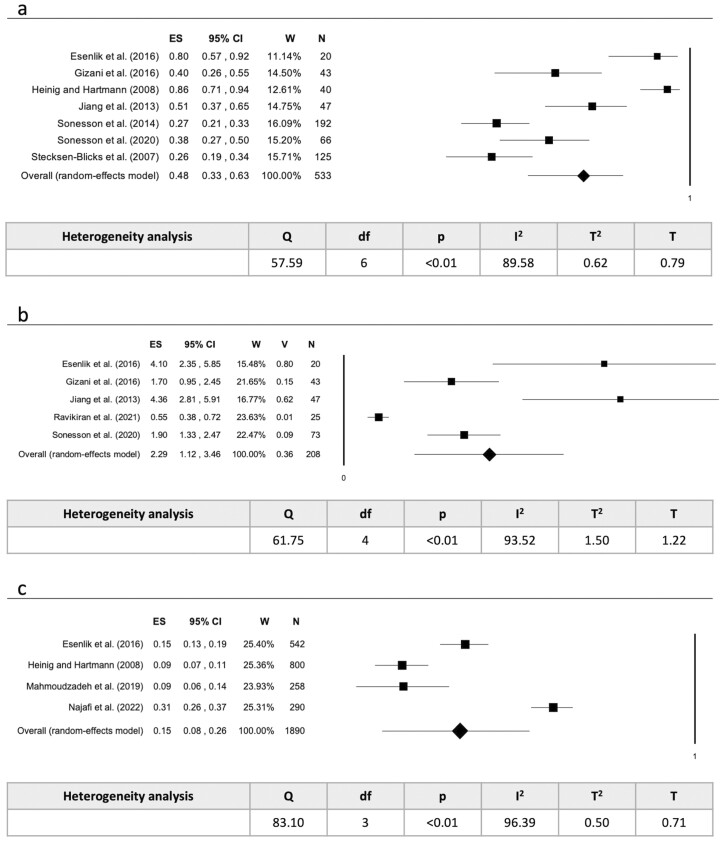
(a) Pooled incidence as percentage per subjects; (b) pooled incidence as mean per subject; (c) pooled incidence as percentage per surface. Legend: ES = Effect Size; CI = Confidence Interval; W = Weight; V = Variance; N = subject/surface; Sig = significance; df = degree of freedom; T = Tau.

The data collected for the meta-analysis were analysed according to the type of outcome (prevalence or incidence), the type of data (percentage or mean), and whether it related to the subject or the tooth surface. Two studies [30, 37] were excluded from the meta-analysis due to data type. Random effects model was used to evaluate the pooled prevalence as percentage per subject (*P* < .01; *I*^2^ = 88.05%) (Fig. 4a), as mean per subject (*P* < .01; *I*^2^ = 97.32%) (Fig. 4b) and as percentage per surface (*P* < .01; *I*^2^ = 99.21%) (Fig. 4c). The pooled incidence has also been assessed as percentage per subject (*P* < .01; *I*^2^ = 89.58%) (Fig. 5a), as mean per subject (*P* < .01; *I*^2^ = 93.52%) (Fig. 5b) and as percentage per surface (*P* < .01; *I*^2^ = 96.39%) (Fig. 5c).

During or at the end of orthodontic treatment, 57.00% of subjects had ICLs, with an average of 2.24 lesions per subject and 22.00% of surfaces affected. Overall, 48.00% of subjects developed new ICLs, with an average of 2.29 new lesions per subject, and 15% of surfaces becoming affected.

**Figure 6.**
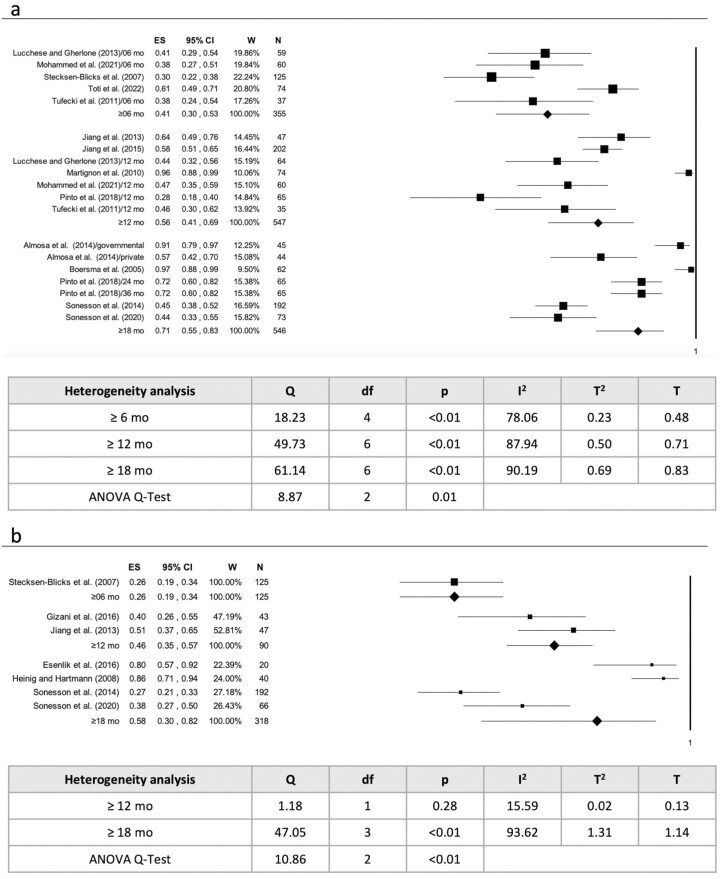
(a) Association between orthodontic treatment duration and ICLs prevalence; (b) association between orthodontic treatment duration and ICLs incidence. Legend: ES = Effect Size; CI = Confidence Interval; W = Weight; V = Variance; N = subject/surface; Sig = significance; df = degree of freedom; T = Tau.

Prevalence and incidence were positively associated with orthodontic treatment duration, with an increase in the number of ICLs as the number of months of treatment increases (*P* = .01 and *P* < .01) (Fig. 6a and b).

No association was found between the age of patients and the number of ICLs.

The funnel plot of publication bias evaluated for each of the six outcomes (prevalence as percentage per subject, as mean per subject, and as percentage per surface; incidence as percentage per subject, as mean per subject, and as percentage per surface) can be retrieved in Supplementary Fig. S1.

## Discussion

### Main results

Despite the growing interest in non-invasive or minimally invasive treatments to prevent, stop, or mask ICLs, there are no recent global data on the incidence or prevalence of ICLs during or following orthodontic treatment with fixed appliances; this study aimed to fill this gap. The current study gathered data from 19 different publications; more than half of the participants with ICLs during or after an orthodontic treatment, with an average of two lesions per subject and a fifth of surfaces affected. Half of the subjects developed new lesions during or after the orthodontic treatment, with an average of two new lesions per subject, and one sixth of the surfaces becoming affected.

The large number of studies reviewed, the analysis of associated risk factors, and the amount of data collected to describe the prevalence and incidence of ICLs associated with orthodontic treatment is among the major strengths of this study, as, to the best of the authors’ knowledge, no study has presented these data to date. Although the high number of included studies and the overall good quality, few high risks of bias studies were included, there was a significant heterogeneity in the collected data, and a very low level of certainty was found. Reported prevalence and incidence data varied widely among the included studies, even in subgroup analyses according to study type, age, and treatment time. Bearing in mind that the type of orthodontics treatment did not vary among the studies representing an inclusion criterion, the high heterogeneity in the results may be explained primarily by the lack of assessment of individual caries risk, the level of oral hygiene of the included subjects, and the location in which the studies were conducted, as caries risk differs among the various countries examined. Furthermore, the prevalence, despite comparable to incidence, should be interpreted with caution due to a possible overestimation of the association between ICLs and orthodontic treatment.

The obtained results are consistent with those of the single published meta-analysis on the topic [21]. However, the current review only contains three [12, 37, 45] of the 14 papers included in the previous meta-analysis. Retrospective studies and studies in which fluoride was administered, in addition to conventional oral hygiene procedures, were excluded, as they could have biased the data gathered, leading to an underestimation of the effect. Furthermore, it would have been interesting to compare the prevalence of ICLs in subjects who had never undergone orthodontic treatment with that found in the present study. However, there is just one meta-analysis on the prevalence of ICLs in the general population, which was carried out on primary teeth, which makes the comparison unfeasible [46]. This situation could be considered a weakness of the revision, as it is not possible to determine how much orthodontic therapy influences the presence of ICLs, although the data provided confirm the relevance of the problem.

### ICLs and subject-related factors

Initial caries lesions are associated with a multifactorial aetiology that may affect the prevalence and incidence. Age has been identified as a factor that could influence the occurrence of ICLs: as age increases, the risk of effect would be reduced as a result of greater efforts to maintain good oral hygiene [21, 47, 48]. However, the results reported in the studies included in this review were inconsistent, and the meta-analysis did not detect a statistically significant association between age and the number of ICLs. The role of sex on the development of ICLs is also unclear. Although poorer oral health status has been described in men than in women [49], only three out of seven studies found a higher number of lesions in men than in women [12, 37, 45].

The oral bacteria count increases fivefold during orthodontic treatment, as maintaining good oral hygiene through flossing, interdental brushing, and proper brushing methods demands greater effort [50]. In tooth areas where there is usually a low risk of caries, as they are easily cleanable, the brackets, bands, and arch wires provide an extra surface for bacterial colonization, increasing plaque development and lesion formation. A significant association between poor oral hygiene and the presence of ICLs has been described [51], but, surprisingly, only six of the included studies confirmed this association [26, 27, 29–31, 37]. Furthermore, the amount of plaque is not directly proportional to the risk of caries, as it is the cariogenic component of plaque that is responsible for caries [52, 53]. However, only one study has examined plaque composition in relation to the presence of ICLs in patients undergoing orthodontic treatment [37].

The greatest number of ICLs were observed on the lateral incisors and maxillary canines, as well as on the maxillary and mandibular premolars and first molars. The previously published meta-analysis reported the same pattern and hypothesized that the presence of small areas of tooth surface between the gingiva and bracket, as found in the upper lateral incisors, may enhance plaque and debris retention, resulting in a greater decalcification. Furthermore, different levels of salivary exposure may help to explain the pattern of ICL formation [21].

### ICLs and fixed orthodontic treatment-related factors

In addition to ICLs, orthodontic treatment, like other interventions, may expose the patient to a range of risks, including speech difficulties, discomfort, periodontal damage, root resorption, pulp necrosis, and temporomandibular disorder. Treatment variables that could have an impact on the risk of adverse effects include appliance type, force vectors, and treatment duration [54]. The study’s findings validated this association, as both the prevalence and incidence of ICLs were positively related with the length of the orthodontic treatment. The longer the appliance is in place, the more plaque accumulates on the teeth, and so ICLs develop. This factor must be carefully considered by the orthodontist before starting treatment, when assessing the patient’s cooperation and motivation as part of the risk/benefit ratio assessment, and during treatment, considering all the risks that an increase in treatment duration would entail.

Caries lesions are mainly the consequence of modifiable factors and several prevention strategies have been proposed [55]. Fluoride applications during orthodontic treatment have been shown to reduce the occurrence of ICLs. However, as the success of home use products is largely dependent on patient compliance, professional use fluoride products would be desirable and should be an integral part of orthodontic treatment along with biofilm management strategies.

## Conclusions

Orthodontic treatment with fixed appliances increases the risk of plaque accumulation and caries development. However, it is necessary to control for all other risk factors in order to assess the true impact of orthodontic treatment on the development of ICLs. Further studies evaluating the incidence of ICLs should be encouraged in which patients are screened for all factors that may influence the caries risk before and during orthodontic treatment, e.g. using standardized tools such as the Cariogram.

## Supplementary Material

cjae008_suppl_Supplementary_Material

## Data Availability

All data generated or analysed during this study are included in this article [and/or] its supplementary material files. Further enquiries can be directed to the corresponding author.
