# Supplementary material for: Distribution of initial caries lesions in relation to fixed orthodontic therapy. A systematic review and meta-analysis
Source: Eur J Orthod. 2024 Feb 22;46(2):cjae008. doi: 10.1093/ejo/cjae008 (PMC10883713; doi:10.1093/ejo/cjae008)

**White spot lesions and fixed orthodontic therapy: incidence and prevalence. A systematic review and meta-analysis**

Supplementary file

**Table S1.** Prisma 2020 Abstract Checklist and Prisma 2020 check-list.

| **Section and Topic** | | **Item #** | | **Checklist item** | **Reported (Yes/No)** |
| --- | --- | --- | --- | --- | --- |
| **TITLE** | | | | |  |
| Title | | 1 | | Identify the report as a systematic review. | Yes |
| **BACKGROUND** | | | | |  |
| Objectives | | 2 | | Provide an explicit statement of the main objective(s) or question(s) the review addresses. | Yes |
| **METHODS** | | | | |  |
| Eligibility criteria | | 3 | | Specify the inclusion and exclusion criteria for the review. | Yes |
| Information sources | | 4 | | Specify the information sources (e.g. databases, registers) used to identify studies and the date when each was last searched. | Yes |
| Risk of bias | | 5 | | Specify the methods used to assess risk of bias in the included studies. | Yes |
| Synthesis of results | | 6 | | Specify the methods used to present and synthesise results. | Yes |
| **RESULTS** | | | | |  |
| Included studies | | 7 | | Give the total number of included studies and participants and summarise relevant characteristics of studies. | Yes |
| Synthesis of results | | 8 | | Present results for main outcomes, preferably indicating the number of included studies and participants for each. If meta-analysis was done, report the summary estimate and confidence/credible interval. If comparing groups, indicate the direction of the effect (i.e. which group is favoured). | Yes |
| **DISCUSSION** | | | | |  |
| Limitations of evidence | | 9 | | Provide a brief summary of the limitations of the evidence included in the review (e.g. study risk of bias, inconsistency and imprecision). | Yes |
| Interpretation | | 10 | | Provide a general interpretation of the results and important implications. | Yes |
| **OTHER** | | | | |  |
| Funding | | 11 | | Specify the primary source of funding for the review. | No |
| Registration | | 12 | | Provide the register name and registration number. | Yes |
| Section and Topic | | Item # | | Checklist item | Location where item is reported |
| **TITLE** | | | | |  |
| Title | 1 | | Identify the report as a systematic review. | | 1 |
| **ABSTRACT** | | | | |  |
| Abstract | 2 | | See the PRISMA 2020 for Abstracts checklist. | | 1 |
| **INTRODUCTION** | | | | |  |
| Rationale | 3 | | Describe the rationale for the review in the context of existing knowledge. | | 2 |
| Objectives | 4 | | Provide an explicit statement of the objective(s) or question(s) the review addresses. | | 2 |
| **METHODS** | | | | |  |
| Eligibility criteria | 5 | | Specify the inclusion and exclusion criteria for the review and how studies were grouped for the syntheses. | | 3-5 |
| Information sources | 6 | | Specify all databases, registers, websites, organisations, reference lists and other sources searched or consulted to identify studies. Specify the date when each source was last searched or consulted. | | 4 |
| Search strategy | 7 | | Present the full search strategies for all databases, registers and websites, including any filters and limits used. | | 4 |
| Selection process | 8 | | Specify the methods used to decide whether a study met the inclusion criteria of the review, including how many reviewers screened each record and each report retrieved, whether they worked independently, and if applicable, details of automation tools used in the process. | | 4 and Table S3 and S4- Supplementary file |
| Data collection process | 9 | | Specify the methods used to collect data from reports, including how many reviewers collected data from each report, whether they worked independently, any processes for obtaining or confirming data from study investigators, and if applicable, details of automation tools used in the process. | | 4-5 |
| Data items | 10a | | List and define all outcomes for which data were sought. Specify whether all results that were compatible with each outcome domain in each study were sought (e.g. for all measures, time points, analyses), and if not, the methods used to decide which results to collect. | | 4-5 |
|  | 10b | | List and define all other variables for which data were sought (e.g. participant and intervention characteristics, funding sources). Describe any assumptions made about any missing or unclear information. | | 5 |
| Study risk of bias assessment | 11 | | Specify the methods used to assess risk of bias in the included studies, including details of the tool(s) used, how many reviewers assessed each study and whether they worked independently, and if applicable, details of automation tools used in the process. | | 4-5 |
| Effect measures | 12 | | Specify for each outcome the effect measure(s) (e.g. risk ratio, mean difference) used in the synthesis or presentation of results. | | 5 |
| Synthesis methods | 13a | | Describe the processes used to decide which studies were eligible for each synthesis (e.g. tabulating the study intervention characteristics and comparing against the planned groups for each synthesis (item #5)). | | 4-5 |
|  | 13b | | Describe any methods required to prepare the data for presentation or synthesis, such as handling of missing summary statistics, or data conversions. | | 4-5 |
|  | 13c | | Describe any methods used to tabulate or visually display results of individual studies and syntheses. | | 4-5 |
|  | 13d | | Describe any methods used to synthesize results and provide a rationale for the choice(s). If meta-analysis was performed, describe the model(s), method(s) to identify the presence and extent of statistical heterogeneity, and software package(s) used. | | 5 |
|  | 13e | | Describe any methods used to explore possible causes of heterogeneity among study results (e.g. subgroup analysis, meta-regression). | | 5 |
|  | 13f | | Describe any sensitivity analyses conducted to assess robustness of the synthesized results. | | 5 |
| Reporting bias assessment | 14 | | Describe any methods used to assess risk of bias due to missing results in a synthesis (arising from reporting biases). | | 5 |
| Certainty assessment | 15 | | Describe any methods used to assess certainty (or confidence) in the body of evidence for an outcome. | | 5-6 |
| **RESULTS** | | | | |  |
| Study selection | 16a | | Describe the results of the search and selection process, from the number of records identified in the search to the number of studies included in the review, ideally using a flow diagram. | | 6, Figure 1,Table S3 and S4- Supplementary file |
|  | 16b | | Cite studies that might appear to meet the inclusion criteria, but which were excluded, and explain why they were excluded. | | 6, Figure 1,Table S3 and S4- Supplementary file |
| Study characteristics | 17 | | Cite each included study and present its characteristics. | | 6-7 |
| Risk of bias in studies | 18 | | Present assessments of risk of bias for each included study. | | 8-9, Figure 3 |
| Results of individual studies | 19 | | For all outcomes, present, for each study: (a) summary statistics for each group (where appropriate) and (b) an effect estimate and its precision (e.g. confidence/credible interval), ideally using structured tables or plots. | | 5-8, Table 2, Figure 4 and 5 |
| Results of syntheses | 20a | | For each synthesis, briefly summarise the characteristics and risk of bias among contributing studies. | | 8-9 |
|  | 20b | | Present results of all statistical syntheses conducted. If meta-analysis was done, present for each the summary estimate and its precision (e.g. confidence/credible interval) and measures of statistical heterogeneity. If comparing groups, describe the direction of the effect. | | 5-9, Table 2, Figure 4 and 5 |
|  | 20c | | Present results of all investigations of possible causes of heterogeneity among study results. | | 5-9, Table 2, Figure 4 and 5 |
|  | 20d | | Present results of all sensitivity analyses conducted to assess the robustness of the synthesized results. | | Figure 4 and 5 |
| Reporting biases | 21 | | Present assessments of risk of bias due to missing results (arising from reporting biases) for each synthesis assessed. | | 8, Figure 3 |
| Certainty of evidence | 22 | | Present assessments of certainty (or confidence) in the body of evidence for each outcome assessed. | | 8, Supplementary file S5-S6 |
| **DISCUSSION** | | | | |  |
| Discussion | 23a | | Provide a general interpretation of the results in the context of other evidence. | | 9-11 |
|  | 23b | | Discuss any limitations of the evidence included in the review. | | 9-11 |
|  | 23c | | Discuss any limitations of the review processes used. | | 9-11 |
|  | 23d | | Discuss implications of the results for practice, policy, and future research. | | 9-11 |
| **OTHER INFORMATION** | | | | |  |
| Registration and protocol | 24a | | Provide registration information for the review, including register name and registration number, or state that the review was not registered. | | 3 |
|  | 24b | | Indicate where the review protocol can be accessed, or state that a protocol was not prepared. | | 3 |
|  | 24c | | Describe and explain any amendments to information provided at registration or in the protocol. | | 3 |
| Support | 25 | | Describe sources of financial or non-financial support for the review, and the role of the funders or sponsors in the review. | | 11 |
| Competing interests | 26 | | Declare any competing interests of review authors. | | 11 |
| Availability of data, code and other materials | 27 | | Report which of the following are publicly available and where they can be found: template data collection forms; data extracted from included studies; data used for all analyses; analytic code; any other materials used in the review. | | Supplementary material |

**Table S2.** Extraction form.

| **Type of teeh included** | **Study design** | **WS lesion at baseline (yes/no)** | **Type of evaluation WS lesion (clinical, photographic…)** | **Type of wsl index (Gorelick, icdas etc)** | **Drying before evaluation (yes/no)** | **Type of orthodontic appliance** | **Duration of orthodontic treatment** | **Other evaluation at baseline (DMFT, oral hygiene, …)** | **Fluoride supplement: has been investigated? (yes/no)** | **Water fluoridation: has been investigated? (yes/no)** | **Incidence or prevalence or both?** | **Primary outcome** | **Secondary outcome** | **Results of primary outcome REFERENCE group** | **Results of primary outcome OTHER group** | **Results of secondary outcome REFERENCE group** | **Results of secondary outcome OTHER group** | **p REFERENCE group** | **p OTHER group** | **p intergroup** |
| --- | --- | --- | --- | --- | --- | --- | --- | --- | --- | --- | --- | --- | --- | --- | --- | --- | --- | --- | --- | --- |
|  |  |  |  |  |  |  |  |  |  |  |  |  |  |  |  |  |  |  |  |  |

**Table S4.** Studies excluded after title and abstract screening.

| **Title** | **Year** | **Journal** | **Authors** | **URL** | **Language** |
| --- | --- | --- | --- | --- | --- |
| Efficacy of CPP-ACP and CPP-ACPF for prevention and remineralization of white spot lesions in orthodontic patients: A systematic review of randomized controlled clinical trials | 2019 | Acta Informatica Medica | Imani, M.M. and Safaei, M. and Afnaniesfandabad, A. and Moradpoor, H. and Sadeghi, M. and Golshah, A. and Sharifi, R. and Mozaffari, H.R. | https://www.embase.com/search/results?subaction=viewrecord&id=L630036785&from=export U2 - L630036785 | English |
| Molar-incisor hypomineralization: an umbrella review. | 2021 | Acta odontologica Scandinavica | Bandeira Lopes L and Machado V and Botelho J and Haubek D | https://pubmed.ncbi.nlm.nih.gov/33524270/ | English |
| Association between orthodontic treatment need and caries experience. | 2011 | Acta odontologica Scandinavica | Borzabadi-Farahani A and Eslamipour F and Asgari I | https://pubmed.ncbi.nlm.nih.gov/20923258/ | English |
| An analysis of the incidence of orthodontic anomalies and caries in the deciduous teeth of children from Zagreb. | 1984 | Acta stomatologica Croatica | MiliciÄ‡ A and Gazi-Coklica V and Hunski M | https://pubmed.ncbi.nlm.nih.gov/6592970/ | Croatian |
| Prevalence of orthodontic anomalies, analysis and evaluation of dental health in three groups of pre-school children in Zadar. | 1990 | Acta stomatologica Croatica | ViskoviÄ‡ R and VujanoviÄ‡ M and BrciÄ‡ V | https://pubmed.ncbi.nlm.nih.gov/2131756/ | Croatian |
| Enamel demineralization following orthodontic treatment. | 1982 | American Journal of Orthodontics | Mizrahi E | <https://pubmed.ncbi.nlm.nih.gov/6984291/> | English |
| Incidence of white spot formation after bonding and banding | 1982 | American Journal of Orthodontics | Gorelick, L. and Geiger, A.M. and Gwinnett, A.J. | <https://www.scopus.com/inward/record.uri?eid=2-s2.0-0020091931&doi=10.1016%2f0002-9416%2882%2990032-X&partnerID=40&md5=d7e934d410b8c10f1a117c2c08a861de> | English |
| Surface distribution of enamel opacities following orthodontic treatment | 1983 | American Journal of Orthodontics | Mizrahi, E. | <https://www.scopus.com/inward/record.uri?eid=2-s2.0-0020841918&doi=10.1016%2fS0002-9416%2883%2990348-2&partnerID=40&md5=328f9ded474a09e3f5f72af6873d8b80> | English |
| Caries and periodontal reactions associated with long-term use of different types of bonded lingual retainers. | 1984 | American journal of orthodontics | Artun J | https://pubmed.ncbi.nlm.nih.gov/6380296/ | English |
| Caries incidence in relation to orthodontic therapy. | 1950 | American journal of orthodontics | DOLCE JJ | https://pubmed.ncbi.nlm.nih.gov/15425631/ | English |
| Cause and prevention of injuries to teeth and supporting structures during orthodontic treatment. | 1976 | American journal of orthodontics | Zachrisson BU | https://pubmed.ncbi.nlm.nih.gov/766644/ | English |
| Modified fluoride toothpaste technique reduces caries in orthodontic patients: A longitudinal, randomized clinical trial. | 2010 | American journal of orthodontics and dentofacial orthopedics | Al Mulla AH and Kharsa SA and Birkhed D | https://pubmed.ncbi.nlm.nih.gov/20816297/ | English |
| Association between incisor irregularity and coronal caries: A population-based study. | 2019 | American journal of orthodontics and dentofacial orthopedics | Alsulaiman AA and Briss DS and Parsi GK and Will LA | https://pubmed.ncbi.nlm.nih.gov/30826040/ | English |
| Effect of methacrylated-based antibacterial monomer on orthodontic adhesive system properties | 2015 | American journal of orthodontics and dentofacial orthopedics | Altmann, A.S. and Collares, F.M. and Ogliari, F.A. and Samuel, S.M. | https://www.embase.com/search/results?subaction=viewrecord&id=L604896722&from=export U2 - L604896722 | English |
| Reduction in incidence of white spot lesions with lingual appliances | 2016 | American journal of orthodontics and dentofacial orthopedics | Dalessandri, D. | https://www.embase.com/search/results?subaction=viewrecord&id=L611483452&from=export U2 - L611483452 | English |
| Effect of a mouth rinse and a high-fluoride toothpaste on caries incidence in orthodontic patients: A randomized controlled trial. | 2022 | American journal of orthodontics and dentofacial orthopedics | EnerbÃ¤ck H and LingstrÃ¶m P and MÃ¶ller M and NylÃ©n C and Ã–dman Bresin C and Ã–stman Ros I and Westerlund A | https://pubmed.ncbi.nlm.nih.gov/35491328/ | English |
| Caries risk after interproximal enamel reduction. | 2006 | American journal of orthodontics and dentofacial orthopedics | Jarjoura K and Gagnon G and Nieberg L | https://pubmed.ncbi.nlm.nih.gov/16849068/ | English |
| Difference in dental lateral arch length between 9-year-olds born in the 1960s and the 1980s. | 2000 | American journal of orthodontics and dentofacial orthopedics | Lindsten R and Ogaard B and Larsson E | https://pubmed.ncbi.nlm.nih.gov/10842109/ | English |
| Development of white spot lesions during orthodontic treatment: perceptions of patients, parents, orthodontists, and general dentists. | 2012 | American journal of orthodontics and dentofacial orthopedics | Maxfield BJ and Hamdan AM and TÃ¼fekÃ§i E and Shroff B and Best AM and Lindauer SJ | https://pubmed.ncbi.nlm.nih.gov/22381494/ | English |
| Effects of fixed-appliance orthodontic treatment on DMF indices. | 1986 | American journal of orthodontics and dentofacial orthopedics | Southard TE and Cohen ME and Ralls SA and Rouse LA | https://pubmed.ncbi.nlm.nih.gov/3461705/ | English |
| Dental health assessed after interproximal enamel reduction: caries risk in posterior teeth. | 2011 | American journal of orthodontics and dentofacial orthopedics | Zachrisson BU and Minster L and Ogaard B and Birkhed D | https://pubmed.ncbi.nlm.nih.gov/21195282/ | English |
| An evaluation of a fluoride-releasing, visible light-activated bonding system for orthodontic bracket placement. | 1989 | American journal of orthodontics and dentofacial orthopedics | Sonis AL and Snell W | <https://pubmed.ncbi.nlm.nih.gov/2650531/> | English |
| Bond failure of a no-mix adhesive during orthodontic treatment. | 2002 | American journal of orthodontics and dentofacial orthopedics | Adolfsson U and Larsson E and Ogaard B | <https://pubmed.ncbi.nlm.nih.gov/12226609/> | English |
| Incidence of caries lesions among patients treated with comprehensive orthodontics | 2011 | American journal of orthodontics and dentofacial orthopedics | Richter, A.E. and Arruda, A.O. and Peters, M.C. and Sohn, W. | <https://www.embase.com/search/results?subaction=viewrecord&id=L361698344&from=export> | English |
| Prevalence of white spot lesions in 19-year-olds: A study on untreated and orthodontically treated persons 5 years after treatment | 1989 | American journal of orthodontics and dentofacial orthopedics | Ogaard, B. | <https://www.embase.com/search/results?subaction=viewrecord&id=L20019912&from=export> | English |
| Validation of caries risk assessment methods in orthodontic patients. | 2020 | American journal of orthodontics and dentofacial orthopedics | Hanna Enerbäck , Peter Lingström , Marie Möller , Cathrine Nylén , Cecilia Ödman Bresin , Ingrid Östman Ros , Anna Westerlund | <https://pubmed.ncbi.nlm.nih.gov/32448565/> | English |
| White-spot lesions during multibracket appliance treatment: A challenge for clinical excellence. | 2011 | American journal of orthodontics and dentofacial orthopedics | Enaia M and Bock N and Ruf S | <https://pubmed.ncbi.nlm.nih.gov/21724067/> | English |
| Role of taste perception in white spot lesion formation during orthodontic treatment | 2019 | Angle Orthodontist | Alanzi, A. and Velissariou, M. and Al-Melh, M.A. and Ferguson, D. and Kavvadia, K. | https://www.scopus.com/inward/record.uri?eid=2-s2.0-85068491933&doi=10.2319%2f091918-680.1&partnerID=40&md5=c5bf2fb5743cba665cad132ee2944599 | English |
| Influence of orthodontic appliance type on salivary parameters during treatment | 2020 | Angle Orthodontist | Dallel, I. and Ben Salem, I. and Merghni, A. and Bellalah, W. and Neffati, F. and Tobji, S. and Mastouri, M. and Ben Amor, A. | https://www.embase.com/search/results?subaction=viewrecord&id=L633898370&from=export U2 - L633898370 | English |
| Glucosidase activity in dental biofilms in adolescent patients with fixed orthodontic appliances - a putative marker for white spot lesions - a clinical exploratory trial. | 2019 | Archives of oral biology | Mikael Sonesson, Gunnel Svensäter , Claes Wickström | <https://pubmed.ncbi.nlm.nih.gov/31004977/> | English |
| Dental emergencies in a university-based pediatric dentistry postgraduate outpatient clinic: a retrospective study. | 2001 | ASDC journal of dentistry for children | Agostini FG and Flaitz CM and Hicks MJ | https://pubmed.ncbi.nlm.nih.gov/11985190/ | English |
| Color changes of post-debonding white spot lesion after microabrasion technique with fluoride and casein phosphopeptide-amorphous calcium phosphate application | 2018 | Asian Journal of Pharmaceutical and Clinical Research | Angriawan, I.P. and Anggani, H.S. and Ismah, N. | https://www.embase.com/search/results?subaction=viewrecord&id=L622882031&from=export U2 - L622882031 | English |
| Prevalence of white spot lesions during clear aligner therapy: a systematic review | 2022 | Australasian Orthodontic Journal | Abay, F. and KutalmÄ±ÅŸ Buyuk, S. and Korkmaz, Y.N. | https://www.scopus.com/inward/record.uri?eid=2-s2.0-85141877519&doi=10.2478%2faoj-2022-0035&partnerID=40&md5=b9c12c3bebd214713dd84ee836e98c22 | English |
| Reducing iatrogenic complications in the treatment of cleft lip and palate | 2019 | Australasian Orthodontic Journal | Savundra, A. and Fricker, J. | https://www.scopus.com/inward/record.uri?eid=2-s2.0-85089471547&partnerID=40&md5=65d15fd5bf9c28756d6e795836927d18 | English |
| White spot lesions in dentistry. Current concepts | 2016 | Avances en Odontoestomatologia | Vargas Sanhueza, J. and Vargas Del Valle, P. and Palomino, H. | https://www.scopus.com/inward/record.uri?eid=2-s2.0-85007329025&partnerID=40&md5=4cea0cf981e7e4ea5c8c989319626b84 | Spanish |
| Chitosan bio-active designer materials and orthodontics: Development and evaluation of novel materials as enamel protective agents | 2016 | Biomaterials and Designer Functional Applications in Oral Cavity |  |  | English |
| Effect of the addition of Chitosan and TiO(2)nanoparticles on antibacterial properties of an orthodontic composite in fixed orthodontic treatment: a randomized clinical trial study. | 2021 | Biomedical physics & engineering express | Farzanegan F and Shahabi M and Niazi AE and Soleimanpour S and Shafaee H and Rangrazi A | https://pubmed.ncbi.nlm.nih.gov/34044375/ | English |
| Efficacy of fluoride varnish for prevention of white spot lesions during orthodontic treatment with fixed appliances: A systematic review study | 2017 | Biomedical Research and Therapy | Rahimi, F. and Sadeghi, M. and Mozaffari, H.R. | https://www.embase.com/search/results?subaction=viewrecord&id=L618219530&from=export U2 - L618219530 | English |
| Distribution of white spot lesions among orthodontic patients attending teaching institutes in Khartoum. | 2017 | BMC oral health | Eltayeb MK and Ibrahim YE and El Karim IA and Sanhouri NM | <https://pubmed.ncbi.nlm.nih.gov/28545439/> | English |
| The prevalence of malocclusion and its association with dental caries among 12-18-year-old disabled adolescents. | 2014 | BMC oral health | Vellappally S and Gardens SJ and Al Kheraif AA and Krishna M and Babu S and Hashem M and Jacob V and Anil S | https://pubmed.ncbi.nlm.nih.gov/25273325/ | English |
| Fluoride-Releasing Materials to Prevent White Spot Lesions around Orthodontic Brackets: A Systematic Review | 2016 | Brazilian Dental Journal | Nascimento, P.L. and Fernandes, M.T. and Figueiredo, F.E. and Faria-E-Silva, A.L. | https://www.embase.com/search/results?subaction=viewrecord&id=L614812872&from=export U2 - L614812872 | English |
| Demineralization of teeth in mouth-breathing patients undergoing maxillary expansion. | 2010 | Brazilian journal of otorhinolaryngology | Bakor SF and Pereira JC and Frascino S and Ladalardo TC and Pignatari SS and Weckx LL | <https://pubmed.ncbi.nlm.nih.gov/21180937/> | English |
| Impact of malocclusion on oral health-related quality of life among schoolchildren. | 2018 | Brazilian oral research | GuimarÃ£es SPA and Jorge KO and Fontes MJF and Ramos-Jorge ML and AraÃºjo CTP and Ferreira EF and MelgaÃ§o CA and Zarzar PM | https://pubmed.ncbi.nlm.nih.gov/30231170/ | English |
| Anticariogenic effect of fluoride-releasing elastomers in orthodontic patients. | 2007 | Brazilian oral research | Miura KK and Ito IY and Enoki C and Elias AM and Matsumoto MA | https://pubmed.ncbi.nlm.nih.gov/17710288/ | English |
| Effects of chlorhexidine varnish on caries during orthodontic treatment: a systematic review and meta-analysis. | 2016 | Brazilian oral research | Okada EM and Ribeiro LN and Stuani MB and Borsatto MC and Fidalgo TK and Paula-Silva FW and KÃ¼chler EC | https://pubmed.ncbi.nlm.nih.gov/27901199/ | English |
| Prevention and treatment of demineralisation during fixed appliance therapy: a review of current methods and future applications | 2013 | British Dental Journal | Chambers, C. and Stewart, S. and Su, B. and Sandy, J. and Ireland, A. | https://www.embase.com/search/results?subaction=viewrecord&id=L603766235&from=export U2 - L603766235 | English |
| Patient safety: reducing the risk of wrong tooth extraction. | 2017 | British dental journal | Cullingham P and Saksena A and Pemberton MN | https://pubmed.ncbi.nlm.nih.gov/28546594/ | English |
| The 1993 national survey of children's dental health. | 1995 | British dental journal | Downer MC | https://pubmed.ncbi.nlm.nih.gov/7599013/ | English |
| Forty years of national surveys: An overview of children's dental health from 1973-2013. | 2015 | British dental journal | Murray JJ and Vernazza CR and Holmes RD | https://pubmed.ncbi.nlm.nih.gov/26404992/ | English |
| Diagnosis and treatment options for anterior white spot lesions | 2020 | British Dental Journal | Sampson, V. and Sampson, A. | https://www.embase.com/search/results?subaction=viewrecord&id=L632986638&from=export U2 - L632986638 | English |
| The influence of fluoride-releasing bonding composites in the development of artificial white spot lesions. An ex vivo study. | 1994 | British journal of orthodontics | Ghani, S.H. and Creanor, S.L. and Luffingham, J.K. and Foye, R.H. | https://www.scopus.com/inward/record.uri?eid=2-s2.0-0028543795&doi=10.1179%2fbjo.21.4.375&partnerID=40&md5=ae1f83d1ae99fb13933d53a56fbbc051 | English |
| Early-life socioeconomic status and malocclusion in adolescents and young adults in Uruguay. | 2018 | Cadernos de saude publica | Goettems ML and Ourens M and Cosetti L and Lorenzo S and Ãlvarez-Vaz R and Celeste RK | https://pubmed.ncbi.nlm.nih.gov/29513860/ | English |
| Effect of an antibacterial varnish on mutans streptococci in plaque from enamel adjacent to orthodontic appliances. | 1995 | Caries research | Twetman S and Hallgren A and Petersson LG | <https://pubmed.ncbi.nlm.nih.gov/7621493/> | English |
| Caries development in children from 2.5 to 3.5 years of age: a longitudinal study. | 1995 | Caries Research | Grindefjord, M. and DahllÃ¶f, G. and ModÃ©er, T. | https://www.embase.com/search/results?subaction=viewrecord&id=L126207139&from=export U2 - L126207139 | English |
| The use of epigallocatechin gallate prevents white spot lesions during orthodontic treatment with fixed appliances | 2020 | Chinese Journal of Tissue Engineering Research | Yang, L.Q. and Xie, L.L. and Liang, J.M. | <https://www.embase.com/search/results?subaction=viewrecord&id=L2004311717&from=export> | Chinese |
| Clinical evaluation of remineralization potential of casein phosphopeptide amorphous calcium phosphate nanocomplexes for enamel decalcification in orthodontics | 2012 | Chinese Medical Journal | Wang, J.-X. and Yan, Y. and Wang, X.-J. | https://www.scopus.com/inward/record.uri?eid=2-s2.0-84872303483&doi=10.3760%2fcma.j.issn.0366-6999.2012.22.020&partnerID=40&md5=d38b091fe0d2432c21cfe11cb966e9f7 | English |
| MI Varnish and MI Paste Plus in a caries prevention and remineralization study: a randomized controlled trial. | 2018 | Clinical oral investigations | Rechmann P and Bekmezian S and Rechmann BMT and Chaffee BW and Featherstone JDB | <https://pubmed.ncbi.nlm.nih.gov/29299732/> | English |
| Effectiveness of Clinpro Tooth me in comparison with MI Varnish with RECALDENT for treatment of white spot lesions: a randomized controlled trial. | 2022 | Clinical Oralal Investigation | Handa A and Chengappa D and Sharma P and Handa JK | https://pubmed.ncbi.nlm.nih.gov/36322154/ | English |
| Effectiveness of remineralizing agents in the prevention and reversal of orthodontically induced white spot lesions: a systematic review and network meta-analysis | 2020 | Clinical Oralal Investigation | Hu, H. and Feng, C. and Jiang, Z. and Wang, L. and Shrestha, S. and Yan, J. and Shu, Y. and Ge, L. and Lai, W. and Hua, F. and Long, H. | https://www.embase.com/search/results?subaction=viewrecord&id=L633186428&from=export U2 - L633186428 | English |
| The lingual enamel morphology and bracket shear bond strength influenced by Nd:YAG laser and aluminum oxide sandblasting preconditioning. | 2021 | Clinical Oralal Investigation | Lopes MS and Pereira DL and de Oliveira Mota CCB and Amaral MM and Zezell DM and Gomes ASL | https://pubmed.ncbi.nlm.nih.gov/32607827/ | English |
| Orthodontic treatment with fixed appliances and biofilm formation--a potential public health threat? | 2014 | Clinical Oralal Investigation | Ren, Y. and Jongsma, M.A. and Mei, L. and van der Mei, H.C. and Busscher, H.J. | https://www.embase.com/search/results?subaction=viewrecord&id=L611680523&from=export U2 - L611680523 | English |
| Influence of a programme for prevention of early childhood caries on early orthodontic treatment needs. | 2020 | Clinical Oralal Investigation | Wagner Y and Knaup I and Knaup TJ and Jacobs C and Wolf M | https://pubmed.ncbi.nlm.nih.gov/32382925/ | English |
| Management of white spot lesions induced during orthodontic treatment with multibracket appliance: a national-based survey | 2022 | Clinical Oralal Investigation | Weyland, M.I. and Jost-Brinkmann, P.-G. and Bartzela, T. | https://www.embase.com/search/results?subaction=viewrecord&id=L637621416&from=export U2 - L637621416 | English |
| Novel Technology for Enamel Remineralization in Artificially Induced White Spot Lesions: In Vitro Study | 2022 | Coatings | Voina Cosma, L.L. and Moldovan, M. and Muntean, A. and Olteanu, C.D. and Chifor, R. and Badea, M.E. | https://www.scopus.com/inward/record.uri?eid=2-s2.0-85138727910&doi=10.3390%2fcoatings12091285&partnerID=40&md5=12546caf13f61dc1da4085265fadd1cf | English |
| Fluorides for preventing early tooth decay (demineralised lesions) during fixed brace treatment | 2019 | Cochrane Database of Systematic Reviews | Benson, P.E. and Parkin, N. and Dyer, F. and Millett, D.T. and Germain, P. | https://www.scopus.com/inward/record.uri?eid=2-s2.0-84977805280&doi=10.1002%2f14651858.CD003809.pub4&partnerID=40&md5=847f8a315b24c293e8237071ece09057 | English |
| Fluorides for the prevention of white spots on teeth during fixed brace treatment. | 2004 | Cochrane database of systematic reviews (Online) | Benson, P.E. and Parkin, N. and Millett, D.T. and Dyer, F.E. and Vine, S. and Shah, A. | https://www.scopus.com/inward/record.uri?eid=2-s2.0-16544379282&partnerID=40&md5=b4544bf54a78564c0e983d3fdd1155d3 | English |
| Fluorides for the prevention of early tooth decay (demineralised white lesions) during fixed brace treatment | 2013 | Cochrane Database Systematic Review | Benson, P.E. and Parkin, N. and Dyer, F. and Millett, D.T. and Furness, S. and Germain, P. | https://www.embase.com/search/results?subaction=viewrecord&id=L620561561&from=export U2 - L620561561 | English |
| Incidence of caries in children of rural and subrural areas in Croatia. | 2008 | Collegium antropologicum | JuriÄ‡ H and KlariÄ‡ T and Zagar M and BukoviÄ‡ D Jr and JankoviÄ‡ B and Spalj S | https://pubmed.ncbi.nlm.nih.gov/18494198/ | English |
| British Association for the Study of Community Dentistry (BASCD) coordinated National Health Service surveys of caries prevalence 1985/6-1995/6. | 1997 | Community dental health | Pitts NB and Evans DJ | https://pubmed.ncbi.nlm.nih.gov/9114552/ | English |
| Malocclusion and orthodontic treatment need measured by the dental aesthetic index and its association with dental caries in Indian schoolchildren. | 2011 | Community dental health | Singh A and Purohit B and Sequeira P and Acharya S and Bhat M | https://pubmed.ncbi.nlm.nih.gov/22320073/ | English |
| Survey of reasons for extraction of permanent teeth in Italy. | 1996 | Community dentistry and oral epidemiology | Angelillo IF and Nobile CG and Pavia M | https://pubmed.ncbi.nlm.nih.gov/8954220/ | English |
| Primary reasons for extraction of permanent teeth in Norway: changes from 1968 to 1988. | 1991 | Community dentistry and oral epidemiology | Klock KS and Haugejorden O | https://pubmed.ncbi.nlm.nih.gov/1764900/ | English |
| Dental and social effects of malocclusion and effectiveness of orthodontic treatment: a strategy for investigation. | 1986 | Community dentistry and oral epidemiology | Shaw WC and Addy M and Dummer PM and Ray C and Frude N | https://pubmed.ncbi.nlm.nih.gov/3485508/ | English |
| The effect of fixed orthodontic treatment on DMFT index and white spot formation | 2014 | Cumhuriyet Dental Journal | Nimet Ünlü, Fatma Betül Kahraman, Said Karabekiroğlu, Zehra İleri | <https://www.scopus.com/inward/record.uri?eid=2-s2.0-84913619856&doi=10.7126%2fcdj.58140.5000011983&partnerID=40&md5=5265637fade55fa28c207d543e792e96> | Turkish |
| Pathophysiology of Demineralization, Part II: Enamel White Spots, Cavitated Caries, and Bone Infection | 2022 | Current Osteoporosis Reports | Roberts, W.E. and Mangum, J.E. and Schneider, P.M. | https://www.embase.com/search/results?subaction=viewrecord&id=L2015028767&from=export U2 - L2015028767 | English |
| Cariologic aspects of orthodontic treatment | 1989 | Den Norske tannlaegeforenings tidende | Ogaard, B. | https://www.scopus.com/inward/record.uri?eid=2-s2.0-0024781968&partnerID=40&md5=f6c4d3c06ba8b6de6ae9abcdf344880f | Norwegian |
| Effect of teledentistry on the oral health status of patients undergoing fixed orthodontic treatment at the first three follow-up visits. | 2021 | Dental and medical problems | Borujeni ES and Sarshar F and Nasiri M and Sarshar S and Jazi L | https://pubmed.ncbi.nlm.nih.gov/34516051/ | English |
| Incidence of caries in dysganthic patients not wearing orthodontic appliances. | 1965 | Dental Cadmos | Luka'cs A and Crosetto E | https://pubmed.ncbi.nlm.nih.gov/5277261/ | Italian |
| Are fluoride releasing dental materials clinically effective on caries control? | 2016 | Dental materials | Cury JA and de Oliveira BH and dos Santos AP and Tenuta LM | https://pubmed.ncbi.nlm.nih.gov/26777115/ | English |
| Review on fluoride-releasing restorative materials--fluoride release and uptake characteristics, antibacterial activity and influence on caries formation. | 2007 | Dental materials | Wiegand A and Buchalla W and Attin T | https://pubmed.ncbi.nlm.nih.gov/16616773/ | English |
| Novel orthodontic cement containing dimethylaminohexadecyl methacrylate with strong antibacterial capability. | 2017 | Dental materials journal | Feng X and Zhang N and Xu HHK and Weir MD and Melo MAS and Bai Y and Zhang K | https://pubmed.ncbi.nlm.nih.gov/28652555/ | English |
| Plaque-removal effect of ultrafine bubble water: Oral application in patients undergoing orthodontic treatment. | 2021 | Dental materials journal | Sueishi N and Ohshima T and Oikawa T and Takemura H and Kasai M and Kitano K and Maeda N and Nakamura Y | https://pubmed.ncbi.nlm.nih.gov/33055432/ | English |
| Reducing white spot lesion incidence during fixed appliance therapy | 2013 | Dental Update | Greene, L.E. and Bearn, D.R. | https://www.embase.com/search/results?subaction=viewrecord&id=L369552490&from=export U2 - L369552490 | English |
| Prevalence of white spot lesions during orthodontic treatment | 2020 | Drug Invention Today | Deepika, V. and Navaneethan, R. | <https://www.embase.com/search/results?subaction=viewrecord&id=L2008424129&from=export> | English |
| A survey on the treatment timing and modalities for white spot lesions among various pediatric dentists in India | 2020 | Drug Invention Today | Obadiah, I. and Jeevanandan, G. and Subramanian, E.M.G. | https://www.scopus.com/inward/record.uri?eid=2-s2.0-85096322601&partnerID=40&md5=011f8b24747be748cf80f7dc4c1d9f64 | English |
| A study of occlusal anomalies and tooth loss in children aged 13-15 years in Nairobi. | 1991 | East African medical journal | Ng'ang'a PM | https://pubmed.ncbi.nlm.nih.gov/1800098/ | English |
| Oral health status and behaviour of Greek patients with cleft lip and palate. | 2009 | European archives of paediatric dentistry | Parapanisiou V and Gizani S and Makou M and Papagiannoulis L | https://pubmed.ncbi.nlm.nih.gov/19627672/ | English |
| Effect of Eugenol on Streptococcus mutans Adhesion on NiTi Orthodontic Wires: In Vitro and in Vivo Conditions | 2021 | European Journal of General Dentistry | Fatene, N. and Mounaji, K. and Soukri, A. | https://www.scopus.com/inward/record.uri?eid=2-s2.0-85125440994&doi=10.1055%2fs-0041-1736372&partnerID=40&md5=26ad469c2ffec3606b28bd00de87827e | English |
| Treatment of white spot lesions post fixed orthodontic therapy | 2020 | European Journal of Molecular and Clinical Medicine | Naveed, N. and Thulasiram, and Sabapathy, K. | https://www.embase.com/search/results?subaction=viewrecord&id=L2010483406&from=export U2 - L2010483406 | English |
| Caries outcomes after orthodontic treatment with fixed appliances: Do lingual brackets make a difference? | 2010 | European Journal of Oral Sciences | van der Veen, M.H. and Attin, R. and Schwestka-Polly, R. and Wiechmann, D. | <https://www.embase.com/search/results?subaction=viewrecord&id=L359081048&from=export> | English |
| Increased caries prevalence in 2.5-year-old children with cleft lip and/or palate. | 1996 | European Journal of Oral Sciences | Bokhout, B. and Hofman, F.X. and van Limbeek, J. and Kramer, G.J. and Prahl-Andersen, B. | https://www.embase.com/search/results?subaction=viewrecord&id=L127223723&from=export U2 - L127223723 | English |
| Caries incidence and oral hygiene during orthodontic treatment | 1971 | European Journal of Oral Sciences | ZAGHRISSON, B.U. and ZACHRISSON, S. | https://www.scopus.com/inward/record.uri?eid=2-s2.0-84984427168&doi=10.1111%2fj.1600-0722.1971.tb02028.x&partnerID=40&md5=89485e12a23585131c573c7429c801f8 | English |
| Prevalence of carious white spots after orthodontic treatment with multibonded appliances | 1986 | European Journal of Orthodontics | Ã…rtun, J. and Brobakken, B.O. | https://www.scopus.com/inward/record.uri?eid=2-s2.0-0022818165&doi=10.1093%2fejo%2f8.4.229&partnerID=40&md5=89d1d462f97415f9a582f263538164fd | English |
| Prevalence of malocclusions in Hungarian adolescents. | 2006 | European journal of orthodontics | GÃ¡bris K and MÃ¡rton S and MadlÃ©na M | https://pubmed.ncbi.nlm.nih.gov/16923783/ | English |
| The incidence of caries and white spot lesions in orthodontically treated adolescents with a comprehensive caries prophylactic regimen--a prospective study. | 2012 | European journal of orthodontics | Hadler-Olsen S and Sandvik K and El-Agroudi MA and Ã˜gaard B | https://pubmed.ncbi.nlm.nih.gov/21750245/ | English |
| Caries incidence in orthodontic patients with high levels of Streptococcus mutans. | 1987 | European journal of orthodontics | LundstrÃ¶m F and Krasse B | https://pubmed.ncbi.nlm.nih.gov/3472889/ | English |
| Monitoring regression of post-orthodontic lesions with impedance spectroscopy: a pilot study. | 2019 | European journal of orthodontics | Mortensen D and Gizani S and Salamara O and Sifakakis I and Twetman S | https://pubmed.ncbi.nlm.nih.gov/30407528/ | English |
| Incidence of filled surfaces from 10-18 years of age in an orthodontically treated and untreated group in Norway. | 1989 | European journal of orthodontics | Ogaard B | https://pubmed.ncbi.nlm.nih.gov/2767142/ | English |
| Dental health, halitosis and mouth breathing in 10-to-15 year old children: A potential connection. | 2019 | European journal of paediatric dentistry | Alqutami J and Elger W and Grafe N and Hiemisch A and Kiess W and Hirsch C | https://pubmed.ncbi.nlm.nih.gov/31850768/ | English |
| Early orthodontic treatment: a new index to assess the risk of malocclusion in primary dentition. | 2014 | European journal of paediatric dentistry | Grippaudo C and Paolantonio EG and Pantanali F and Antonini G and Deli R | https://pubmed.ncbi.nlm.nih.gov/25517589/ | English |
| Caries experience, oral disorders, oral hygiene practices and socio-demographic characteristics of autistic children. | 2019 | European journal of paediatric dentistry | Kuter B and Guler N | https://pubmed.ncbi.nlm.nih.gov/31489825/ | English |
| Oral health and malocclusion in 10-to-11 years-old children in southern Italy. | 2009 | European journal of paediatric dentistry | Migale D and Barbato E and BossÃ¹ M and Ferro R and Ottolenghi L | https://pubmed.ncbi.nlm.nih.gov/19364240/ | English |
| Is there an association between various aspects of oral health in Southern Italy children? An epidemiological study assessing dental decays, periodontal status, malocclusions and temporomandibular joint function. | 2018 | European journal of paediatric dentistry | Paduano S and Rongo R and Bucci R and Aiello D and Carvelli G and Ingenito A and Cantile T and Ferrazzano GF | https://pubmed.ncbi.nlm.nih.gov/30063147/ | English |
| Prevalence and determinants of oral impacts on daily performance: results from a survey among school children in Italy. | 2010 | European journal of public health | Bianco A and Fortunato L and Nobile CG and Pavia M | https://pubmed.ncbi.nlm.nih.gov/19892850/ | English |
| Do additional high-fluoride interventions among low caries prevalence orthodontic cases using fixed appliances reduce caries incidence? | 2022 | Evidence-based dentistry | Flores-Mir C | <https://pubmed.ncbi.nlm.nih.gov/36151278/> | English |
| Fluoride varnish reduces white spot lesions during orthodontic treatment. | 2008 | Evidence-based dentistry | Shafi I | <https://pubmed.ncbi.nlm.nih.gov/18927569/> | English |
| Some evidence that fluoride during orthodontic treatment reduces occurrence and severity of white spot lesions: Is fluoride effective in preventing white spot lesions during orthodontic treatment? | 2004 | Evidence-Based Dentistry | Kalha, A. | https://www.scopus.com/inward/record.uri?eid=2-s2.0-34447128607&doi=10.1038%2fsj.ebd.6400298&partnerID=40&md5=cf807f1d6028935ceb81e39f5da4f78c | English |
| Topical fluorides and decalcification around fixed orthodontic appliances: Which topical fluoride preparations are best able to prevent decalcification around fixed orthodontic appliances? | 2006 | Evidence-Based Dentistry | Kalha, A.S. | https://www.scopus.com/inward/record.uri?eid=2-s2.0-34748845509&doi=10.1038%2fsj.ebd.6400398&partnerID=40&md5=a3c1deaff2969ced089f8349ecdb9411 | English |
| Orthodontic anomalies in adolescents. | 2000 | Fogorvosi szemle | GÃ¡bris K and MÃ¡rton S and MadlÃ©na M | https://pubmed.ncbi.nlm.nih.gov/11147181/ | Hungarian |
| Prevalence of dental caries in adolescents wearing the Alexander-type fixed orthodontic device. | 2001 | Fogorvosi szemle | VÃ©gh A and Zeisel M and Patthy A | https://pubmed.ncbi.nlm.nih.gov/11757329/ | English |
| Study on the caries incidence in 6-year molars from the orthodontic viewpoint. | 1965 | Fortschritte der Kieferorthopadie | Gaertner K | https://pubmed.ncbi.nlm.nih.gov/5216307/ | German |
| Oral Microbiota Changes during Orthodontic Treatment | 2022 | Frontiers in Bioscience - Elite | Santonocito, S. and Polizzi, A. | https://www.scopus.com/inward/record.uri?eid=2-s2.0-85138411758&doi=10.31083%2fj.fbe1403019&partnerID=40&md5=10947a2d9349a1d6fca2e8109dcb04e6 | English |
| Enamel resistance in children with malocclusions. | 2020 | Georgian medical news | Smolyar N and Lesitskiy M and Bezvushko E and Fur N and Hordon-Zhura H | https://pubmed.ncbi.nlm.nih.gov/33130643/ | English |
| Oral microbiota carriage in patients with multibracket appliance in relation to the quality of oral hygiene. | 2016 | Head & face medicine | Klaus K and Eichenauer J and Sprenger R and Ruf S | https://pubmed.ncbi.nlm.nih.gov/27793169/ | English |
| Comparison of potential long-term costs for preventive dentistry treatment of post-orthodontic labial versus lingual enamel cavitations and esthetically relevant white-spot lesions: a simulation study with different scenarios. | 2019 | Head & face medicine | KnÃ¶sel M and Vogel Alvarez R and Blanck-Lubarsch M and Helms HJ | https://pubmed.ncbi.nlm.nih.gov/31399113/ | English |
| The factors that influence oral health-related quality of life in 15-year-old children. | 2018 | Health and quality of life outcomes | Sun L and Wong HM and McGrath CPJ | https://pubmed.ncbi.nlm.nih.gov/29347943/ | English |
| Prevalence of malocclusion among 5 387 12- to 14-year-old adolescents in Jiangxi province, China: an epidemiological study. | 2019 | West China journal of stomatology | Xu TT and Zeng LW and Wen JQ and Wan L and Ou XY | https://pubmed.ncbi.nlm.nih.gov/31721505/ | chi |
| Salivary flow rate, pH and buffering capacity in patients undergoing fixed orthodontic treatment - A prospective study. | 2019 | Indian journal of dental research | Anu V and Madan Kumar PD and Shivakumar M | https://pubmed.ncbi.nlm.nih.gov/31745047/ | English |
| Caries prevalence, oral hygiene and orthodontic status of Saudi Bedouin children. | 2001 | Indian journal of dental research | Wyne A and al-Dlaigan Y and Khan N | https://pubmed.ncbi.nlm.nih.gov/11987657/ | English |
| An estimation of blood glucose level in children with early childhood caries-a retrospective study | 2020 | Indian Journal of Forensic Medicine & Toxicology | Iyer, P.K. and Mani, G. and Sarvana Dinesh, S.P. | https://www.embase.com/search/results?subaction=viewrecord&id=L2005817233&from=export U2 - L2005817233 | English |
| Comparison of casein phosphopeptide amorphous calcium phosphate fluoride and fluoride varnish on remineralization of early caries lesions around orthodontic brackets | 2021 | Indian Journal of Forensic Medicine & Toxicology | Mollabashi, V. and Irani, S. and Kazemisaleh, A. and Gharagozlou, S. and Seyedtabib, M. | https://www.embase.com/search/results?subaction=viewrecord&id=L2007506883&from=export U2 - L2007506883 | English |
| Prevalence of caries and malocclusion in an indigenous population in Chiapas, Mexico. | 2015 | International dental journal | Aamodt K and Reyna-Blanco O and Sosa R and Hsieh R and De la Garza Ramos M and Garcia Martinez M and Orellana MF | https://pubmed.ncbi.nlm.nih.gov/26382724/ | English |
| Reasons for extraction in a group of Libyan patients. | 2011 | International dental journal | Byahatti SM and Ingafou MS | https://pubmed.ncbi.nlm.nih.gov/21851351/ | English |
| Dental health in Malaysia. | 1984 | International dental journal | Majid ZA | https://pubmed.ncbi.nlm.nih.gov/6597132/ | English |
| Oral health in South Africa. | 2004 | International dental journal | van Wyk PJ and van Wyk C | https://pubmed.ncbi.nlm.nih.gov/15631099/ | English |
| Effect of fluoridated toothpaste on white spot lesions in postorthodontic patients. | 2013 | International journal of clinical pediatric dentistry | Agarwal A and Pandey H and Pandey L and Choudhary G | https://pubmed.ncbi.nlm.nih.gov/25206198/ | English |
| The antimicrobial effect of chlorhexidine varnish on mutans streptococci in patients with fixed orthodontic appliances: a systematic review of clinical efficacy. | 2016 | International journal of dental hygiene | Tang X and Sensat ML and Stoltenberg JL | https://pubmed.ncbi.nlm.nih.gov/26257398/ | English |
| The Effect of Regime Oral-Hygiene Intervention on the Incidence of New White Spot Lesions in Teenagers Treated with Fixed Orthodontic Appliances. | 2020 | International journal of environmental research and public health | Urszula Kozak, Anna Sekowska, Renata Chałas | <https://pubmed.ncbi.nlm.nih.gov/33348756/> | English |
| New Method of Avoiding Underestimation of Caries Incidence and Its Association with Possible Risk Factors in Japanese University Students: A Prospective Cohort Study. | 2022 | International journal of environmental research and public health | Ekuni D and Toyama N and Iwasaki Y and Morita M | https://pubmed.ncbi.nlm.nih.gov/35206677/ | English |
| Oral Health in 12- and 15-Year-Old Children in Serbia: A National Pathfinder Study. | 2022 | International journal of environmental research and public health | Peric T and Campus G and Markovic E and Petrovic B and Soldatovic I and Vukovic A and Kilibarda B and Vulovic J and Markovic J and Markovic D | https://pubmed.ncbi.nlm.nih.gov/36231568/ | English |
| Effectiveness of a preventive program based on caries risk assessment and recall intervals on the incidence and regression of initial caries lesions in children | 2015 | International Journal of Paediatric Dentistry | Abanto, J. and Celiberti, P. and Braga, M.M. and Vidigal, E.A. and Cordeschi, T. and Haddad, A.E. and BÃ¶necker, M. | https://www.embase.com/search/results?subaction=viewrecord&id=L614555821&from=export U2 - L614555821 | English |
| Influence of salivary parameters in the caries development in orthodontic patients-an observational clinical study. | 2017 | International journal of paediatric dentistry | Cardoso AA and Lopes LM and Rodrigues LP and Teixeira JJ and Steiner-Oliveira C and Nobre-Dos-Santos M | https://pubmed.ncbi.nlm.nih.gov/28247450/ | English |
| Oral health-related quality of life in 6- to 12-year-old schoolchildren in Spain. | 2016 | International journal of paediatric dentistry | Montero J and Rosel E and Barrios R and LÃ³pez-Valverde A and Albaladejo A and Bravo M | https://pubmed.ncbi.nlm.nih.gov/26371614/ | English |
| Prevalence of white spot lesions in patients treated with fixed orthodontic appliances | 2020 | International Journal of Pharmaceutical Sciences and Research (IJPSR) | Bano, A.M. and Srirengalakshmi, | <https://www.embase.com/search/results?subaction=viewrecord&id=L2005580929&from=export> | English |
| White spot lesions in orthodontic-a literature review | 2020 | International Journal of Pharmaceutical Sciences and Research (IJPSR) | Hemashree, J. and Jain, R.K. | https://www.embase.com/search/results?subaction=viewrecord&id=L2005584521&from=export U2 - L2005584521 | English |
| Awareness of whitespot lesions in orthodontic patients among dental students | 2020 | International Journal of Pharmaceutical Sciences and Research (IJPSR) | Sowmya, G.S. and Navaneethan, R. and Malaiappan, S. | https://www.embase.com/search/results?subaction=viewrecord&id=L2005254834&from=export U2 - L2005254834 | English |
| Decalcification and bond failure rate in resin modified glass ionomer cement versus conventional composite for orthodontic bonding: A systematic review & meta-analysis | 2020 | International Orthodontics | Khan, A.R. and Fida, M. and Gul, M. | https://www.embase.com/search/results?subaction=viewrecord&id=L630465085&from=export U2 - L630465085 | English |
| Preventive and interceptive orthodontic treatment needs of an inner-city group of 6- and 9-year-old Canadian children. | 2005 | Journal (Canadian Dental Association) | Karaiskos N and Wiltshire WA and Odlum O and Brothwell D and Hassard TH | https://pubmed.ncbi.nlm.nih.gov/16271161/ | English |
| White spot lesions in orthodontics: Prevention and treatment. a descriptive review | 2021 | Journal of Biological Regulators and Homeostatic Agents | Marinelli, G. and Inchingolo, A.D. and Inchingolo, A.M. and Malcangi, G. and Limongelli, L. and Montenegro, V. and Coloccia, G. and Laudadio, C. and Patano, A. and Inchingolo, F. and Bordea, I.R. and Scarano, A. and Lucchina, A.G. and Lorusso, F. and Venere, D.D. and Laforgia, A. and Dipalma, G. | https://www.embase.com/search/results?subaction=viewrecord&id=L2014062595&from=export U2 - L2014062595 | English |
| Multivariate analysis of surface physico-chemical properties controlling biofilm formation on orthodontic adhesives prior to and after fluoride and chlorhexidine treatment. | 2006 | Journal of biomedical materials research. Part B, Applied biomaterials | Chin MY and Sandham A and Pratten J and van der Mei HC and Busscher HJ | https://pubmed.ncbi.nlm.nih.gov/16470828/ | English |
| White spot lesions on teeth during or postorthodontic treatment: A knowledge based cross-sectional survey of dentists | 2021 | Journal of Clinical and Diagnostic Research | Nayak, U.A. and Nawawi, E.Y. and Bazuhair, L.M. and Nasser, M.N. and Sait, R.K. and Nayak, P.A. | https://www.embase.com/search/results?subaction=viewrecord&id=L2014855259&from=export U2 - L2014855259 | English |
| Prevalence of oral microbial flora during orthodontic space closure: A pilot study | 2020 | Journal of Clinical and Diagnostic Research | Selvaraj, A. and Felicita, S. and Girija, S. | https://www.embase.com/search/results?subaction=viewrecord&id=L2010115840&from=export U2 - L2010115840 | English |
| Periodontal conditions in Swiss army recruits: a comparative study between the years 1985, 1996 and 2006. | 2007 | Journal of clinical periodontology | RÃ¶thlisberger B and Kuonen P and Salvi GE and Gerber J and Pjetursson BE and AttstrÃ¶m R and Joss A and Lang NP | https://pubmed.ncbi.nlm.nih.gov/17850604/ | English |
| Application of light-cure resin-modified glass ionomer cement in orthodontic practice | 2008 | Journal of Clinical Rehabilitative Tissue Engineering Research | Shan, L.-H. and Cui, Z.-Q. and Shen, Q.-H. and Gao, Q. and Qiu, Z.-X. | <https://www.scopus.com/inward/record.uri?eid=2-s2.0-42549125176&partnerID=40&md5=78a84f8e80a74129475d894e047f3c34> | English |
| Prevalence of white spot lesion in a section of Indian population undergoing fixed orthodontic treatment: An in vivo assessment using the visual International Caries Detection and Assessment System II criteria | 2012 | Journal of Conservative Dentistry | Sagarika, N. and Suchindran, S. and Loganathan, S.C. and Gopikrishna, V. | <https://www.scopus.com/inward/record.uri?eid=2-s2.0-84859806964&doi=10.4103%2f0972-0707.94572&partnerID=40&md5=fd3f02252f047899fdc581c4aae4a7a5> | English |
| Comparison of Streptococcus mutans concentrations in non-banded and banded orthodontic patients. | 1981 | Journal of dental research | Corbett JA and Brown LR and Keene HJ and Horton IM | https://pubmed.ncbi.nlm.nih.gov/6946108/ | English |
| Detecting white spot lesions on dental photography using deep learning: A pilot study | 2021 | Journal of Dentistry | Askar, H. and Krois, J. and Rohrer, C. and Mertens, S. and Elhennawy, K. and Ottolenghi, L. and Mazur, M. and Paris, S. and Schwendicke, F. | https://www.embase.com/search/results?subaction=viewrecord&id=L634396714&from=export U2 - L634396714 | English |
| Protein-repellent and antibacterial effects of a novel polymethyl methacrylate resin. | 2018 | Journal of dentistry | Cao L and Xie X and Wang B and Weir MD and Oates TW and Xu HHK and Zhang N and Bai Y | https://pubmed.ncbi.nlm.nih.gov/30248381/ | English |
| Synergy of brushing mode and antibacterial use on in vivo biofilm formation. | 2015 | Journal of dentistry | Jongsma MA and van de Lagemaat M and Busscher HJ and Geertsema-Doornbusch GI and Atema-Smit J and van der Mei HC and Ren Y | https://pubmed.ncbi.nlm.nih.gov/26260976/ | English |
| Effectiveness of professional fluorides against enamel white spot lesions during fixed orthodontic treatment: A systematic review and meta-analysis | 2019 | Journal of Dentistry | Sardana, D. and Zhang, J. and Ekambaram, M. and Yang, Y. and McGrath, C.P. and Yiu, C.K.Y. | https://www.embase.com/search/results?subaction=viewrecord&id=L626700141&from=export U2 - L626700141 | English |
| Efficacy of Glass Ionomer Cements for Prevention of White Spot Lesions During Orthodontic Banding: A Randomized Clinical Trial. | 2015 | Journal of dentistry (Tehran, Iran) | Fallahinejad Ghajari M and Eslamian L and Naji Rad A and Morovati SP | https://pubmed.ncbi.nlm.nih.gov/27559351/ | English |
| Association of Candida Species Isolated From the Dental Plaque of HIV-infected Children and Prevalence of Early Carious Lesions | 2016 | Journal of Dentistry for Children | Rosa Oliveira, C.A. and Charone, S. and de AraÃºjo Soares, R.M. and Portela, M.B. and de ArÃ¡ujo Castro, G.F. | https://www.embase.com/search/results?subaction=viewrecord&id=L620575610&from=export U2 - L620575610 | English |
| White spot lesions in orthodontic patients: An expert opinion | 2019 | Journal of International Oral Health | Karad, A. and Dhole, P. and Juvvadi, S. and Joshi, S. and Gupta, A. | https://www.scopus.com/inward/record.uri?eid=2-s2.0-85071288965&doi=10.4103%2fjioh.jioh-129-19&partnerID=40&md5=1204eef3eb545e979df40a2331384692 | English |
| Effect of different white-spot lesion treatment on orthodontic shear strength and enamel morphology: In vitro study | 2020 | Journal of International Oral Health | Triwardhani, A. and Budipramana, M. and Sjamsudin, J. | https://www.scopus.com/inward/record.uri?eid=2-s2.0-85083264067&doi=10.4103%2fjioh.jioh_206_19&partnerID=40&md5=6e380b3c306fc9d3560a75edf8968379 | English |
| Critical evaluation of incidence and prevalence of white spot lesions during fixed orthodontic appliance treatment: A meta-analysis. | 2015 | Journal of International Society of Preventive & Community Dentistry | Sundararaj D and Venkatachalapathy S and Tandon A and Pereira A | https://pubmed.ncbi.nlm.nih.gov/26759794/ | English |
| Evaluation of the efficacy of fluoride varnish on enamel demineralization in orthodontic patients: A split-mouth clinical trial | 2013 | Journal of Medical Sciences (Faisalabad) | Khan, R. and Antony, V.V. | https://www.scopus.com/inward/record.uri?eid=2-s2.0-84877303002&doi=10.3923%2fjms.2013.146.150&partnerID=40&md5=52f7754db01010ec74d557ec5f3497ef | English |
| Antiadherence and antimicrobial properties of silver nanoparticles against streptococcus mutans on brackets and wires used for orthodontic treatments | 2018 | Journal of Nanomaterials | Espinosa-CristÃ³bal, L.F. and LÃ³pez-Ruiz, N. and Cabada-TarÃ­n, D. and Reyes-LÃ³pez, S.Y. and Zaragoza-Contreras, A. and Constandse-CortÃ©z, D. and DonohuÃ©-Cornejo, A. and Tovar-Carrillo, K. and Cuevas-GonzÃ¡lez, J.C. and Kobayashi, T. | https://www.scopus.com/inward/record.uri?eid=2-s2.0-85059068991&doi=10.1155%2f2018%2f9248527&partnerID=40&md5=89332086b1594996a89aa167b2ddc4e7 | English |
| Periodontal health in teenagers treated with removable aligners and fixed orthodontic appliances | 2015 | Journal of Orofacial Orthopedics | Abbate, G.M. and Caria, M.P. and Montanari, P. and Mannu, C. and OrrÃ¹, G. and Caprioglio, A. and Levrini, L. | https://www.scopus.com/inward/record.uri?eid=2-s2.0-84930198054&doi=10.1007%2fs00056-015-0285-5&partnerID=40&md5=bbb5a2811ae1480bdd2c2d87771a4601 | English |
| Orofacial findings in conjunction with infantile cerebral paralysis in adults of two different age groups--a cross-sectional study. | 2008 | Journal of Orofacial Orthopedics | Asdaghi Mamaghani SM and Bode H and Ehmer U | https://pubmed.ncbi.nlm.nih.gov/18797829/ | English |
| The prevalence of approximal caries in patients after fixed orthodontic treatment and in untreated subjects: a retrospective, cross-sectional study on bitewing radiographs. | 2013 | Journal of Orofacial Orthopedics | Baumgartner S and Menghini G and Imfeld T | https://pubmed.ncbi.nlm.nih.gov/23307177/ | English |
| Structural color changes in permanent enamel of patients with cleft lip and palate: a case-control study. | 2016 | Journal of Orofacial Orthopedics | Kulas A and Illge C and Bekes K and Eckert AW and Fuhrmann RA and Hirsch C | https://pubmed.ncbi.nlm.nih.gov/26744208/ | English |
| Effects of nursing bottle misuse on oral health. Prevalence of caries, tooth malalignments and malocclusions in North-German preschool children. | 2008 | Journal of Orofacial Orthopedics | Robke FJ | https://pubmed.ncbi.nlm.nih.gov/18213457/ | English |
| White spot lesions during orthodontic clear aligner therapy: A scoping review | 2022 | Journal of Orthodontic Science | Bisht, S. and Khera, A. and Raghav, P. | https://www.scopus.com/inward/record.uri?eid=2-s2.0-85131794259&doi=10.4103%2fjos.jos_170_21&partnerID=40&md5=5f201651474135a4003945ae47c5994e | English |
| Fluorides, orthodontics and demineralization: a systematic review. | 2005 | Journal of Orthodontics | Benson, P.E. and Shah, A.A. and Millett, D.T. and Dyer, F. and Parkin, N. and Vine, R.S. | https://www.embase.com/search/results?subaction=viewrecord&id=L41066123&from=export U2 - L41066123 | English |
| Association between occlusal anomalies and dental caries in 3- to 5 year-old Brazilian children. | 2011 | Journal of orthodontics | Marquezan M and Marquezan M and Faraco-Junior IM and Feldens CA and Kramer PF and Ferreira SH | https://pubmed.ncbi.nlm.nih.gov/21367823/ | English |
| Influence of increased body mass index on orthodontic tooth movement and related parameters in children and adolescents: A systematic review of longitudinal controlled clinical studies. | 2019 | Journal of orthodontics | Michelogiannakis D and Rossouw PE and Khan J and Akram Z and Menenakos E and Javed F | https://pubmed.ncbi.nlm.nih.gov/31522589/ | English |
| Can oral health-related quality of life measures substitute for normative needs assessments in 11 to 12-year-old children? | 2006 | Journal of public health dentistry | Tsakos G and Gherunpong S and Sheiham A | https://pubmed.ncbi.nlm.nih.gov/17225821/ | English |
| Balancing dental service requirements and supplies: epidemiologic and demographic evidence. | 1990 | Journal of the American Dental Association (1939) | Douglass CW and Furino A | https://pubmed.ncbi.nlm.nih.gov/2229736/ | English |
| Baby-Risk of malocclusion assessment index: An assessment tool for preventive orthodontic treatment needs in a selected population of children in southeast of Iran. | 2021 | Journal of the Indian Society of Pedodontics and Preventive Dentistry | Jahanimoghadam F and Nikzad S and Ahmadipour H and Sadeghi S and Aftabi R | https://pubmed.ncbi.nlm.nih.gov/33885384/ | English |
| An audit of the caries status of patients about to start orthodontic treatment. | 2011 | Journal of the Irish Dental Association | Meade MJ and Millett DT | https://pubmed.ncbi.nlm.nih.gov/21830357/ | English |
| Prevalence of white spot lesions and risk factors associated with the COVID-19 pandemic | 2022 | Journal of the World Federation of Orthodontists | Burnheimer, J.M. and Serio, C.G. and Loo, B.H. and Hartsock, L.A. | <https://www.embase.com/search/results?subaction=viewrecord&id=L2018394742&from=export> | English |
| Incidence of white spot lesions during orthodontic clear aligner therapy | 2017 | Journal of the World Federation of Orthodontists | Azeem, M. and Ul Hamid, W. | <https://www.scopus.com/inward/record.uri?eid=2-s2.0-85029009768&doi=10.1016%2fj.ejwf.2017.07.001&partnerID=40&md5=e8ffd3d4584c42bffc27d2f21b6a7a79> | English |
| Management of ectopically erupting permanent molars in a seven-year-old girl: A case report. | 2021 | JPMA. The Journal of the Pakistan Medical Association | Meng M and Zhou X and Zhang Q and Zou J | https://pubmed.ncbi.nlm.nih.gov/35150544/ | English |
| Enamel demineralization in orthodontics. Systematic use of fluoride in prevention and treatment. | 2012 | Les demineralisations amelaires en orthodontie. Utilisation raisonnee du fluor dans la prevention et le traitement. | Bahoum, A. and Bahije, L. and Zaoui, F. | https://www.embase.com/search/results?subaction=viewrecord&id=L366352134&from=export U2 - L366352134 | German |
| White spot lesions in patients with orthodontic treatment. Literature review | 2021 | Lesiones de mancha blanca en pacientes con tratamiento de ortodoncia. Revision de la Literatura | Sanchez-Tito, M.A. and Jon, L.Y.T.C. | https://www.embase.com/search/results?subaction=viewrecord&id=L2011760090&from=export U2 - L2011760090 | Spanish |
| Anti-Adherence and Antimicrobial Activities of Silver Nanoparticles against Serotypes C and K of Streptococcus mutans on Orthodontic Appliances. | 2022 | Medicina (Kaunas, Lithuania) | Nafarrate-Valdez RA and MartÃ­nez-MartÃ­nez RE and Zaragoza-Contreras EA and Ãyala-Herrera JL and DomÃ­nguez-PÃ©rez RA and Reyes-LÃ³pez SY and Donohue-Cornejo A and Cuevas-GonzÃ¡lez JC and Loyola-RodrÃ­guez JP and Espinosa-CristÃ³bal LF | https://pubmed.ncbi.nlm.nih.gov/35888596/ | English |
| Oral health in 6-year-old schoolchildren from Berisso, Argentina: falling far short of WHO goals. | 2010 | Medicina oral, patologia oral y cirugia bucal | Llompart G and Marin GH and Silberman M and Merlo I and Zurriaga O | https://pubmed.ncbi.nlm.nih.gov/19680177/ | English |
| Current treatment modalities of orthodontically induced white spot lesions and their outcome. A literature review | 2019 | Medicine and Pharmacy Reports | Cosma, L.L. and Åžuhani, R.D. and MesaroÅŸ, A. and Badea, M.E. | https://www.embase.com/search/results?subaction=viewrecord&id=L627546003&from=export U2 - L627546003 | English |
| A descriptive study of malocclusion in Malian, Cuban and Venezuelan 5-9 year-old children. | 2014 | Medwave | MacÃ­as R and MacÃ­as C and Quesada L and Paneque M | https://pubmed.ncbi.nlm.nih.gov/25383985/ | Spanish |
| Comparative Efficacy of MI Paste Plus and Fluoride Varnish Remineralizing Agents for Preventing White Spot Lesions in Patients on Fixed Orthodontic Treatment: A Clinical Trial | 2022 | Middle East Journal of Rehabilitation and Health Studies | Kaveh, S. and Karamifar, A. and Kousha, S. and Samani, Y. and Azarbyjani, A. and Mirmohammadkhani, M. | <https://www.scopus.com/inward/record.uri?eid=2-s2.0-85124880932&doi=10.5812%2fmejrh.118635&partnerID=40&md5=85d450b6fac264c088edfaa1d0742d82> | English |
| Mineral content identification of white spot lesions around orthodontic brackets following the use of different concentrations of nano-hydroxyapatite mouthwash and fluoride gel | 2020 | Middle East Journal of Rehabilitation and Health Studies | Talaei, R. and Hoseini, Z. and Ghorbani, R. and Ameli, N. | https://www.scopus.com/inward/record.uri?eid=2-s2.0-85090673580&doi=10.5812%2fmejrh.96516&partnerID=40&md5=4b0a5b0d710fdf6e66412030de346bc9 | English |
| Oral tissues and orthodontic treatment: common side effects. | 2013 | Minerva stomatologica | Farronato G and Giannini L and Galbiati G and Cannalire P and Martinelli G and Tubertini I and Maspero C | https://pubmed.ncbi.nlm.nih.gov/24270203/ | English |
| Early childhood caries. Oral health survey in kindergartens of the 19th district in Rome. | 2004 | Minerva stomatologica | Panetta F and Dall'Oca S and Nofroni I and Quaranta A and Polimeni A and Ottolenghi L | https://pubmed.ncbi.nlm.nih.gov/15894942/ | English |
| Caries or fluoridation--a comparison between Hamburg and Vordingborg (Denmark). | 1977 | Monatsschrift fur Kinderheilkunde | Hein G | https://pubmed.ncbi.nlm.nih.gov/840228/ | German |
| Incidence of enamel fissures in a group of orthodontic patients. In vivo study. | 1990 | Mondo ortodontico | Colangelo GG and Festa F and Buffone P | https://pubmed.ncbi.nlm.nih.gov/2280787/ | Italian |
| Pattern of malocclusion and caries experience in unrepaired cleft lip and palate patients in Enugu. | 2020 | Nigerian journal of clinical practice | Okoye LO and Onah II and Ekwueme OC and Agu KA | https://pubmed.ncbi.nlm.nih.gov/31929208/ | English |
| Effects of first permanent molar extractÄ±on on space changes observed in the dental arch using data mining method. | 2019 | Nigerian journal of clinical practice | Serindere G and Bolgul B and Parlar T and Cosgun A | https://pubmed.ncbi.nlm.nih.gov/31293258/ | English |
| Demineralization preventive practices among Nigerian orthodontists-An evidence-based approach? | 2020 | Nigerian journal of clinical practice | Umeh OD and Utomi IL and Ndukwe AN and Izuka M | https://pubmed.ncbi.nlm.nih.gov/32367863/ | English |
| Effect of chlorhexidine varnish and fluoride varnish on white spot lesions in orthodontic patients-a systematic review | 2021 | Open Dentistry Journal | Govindaraj, A. and Dinesh, S.P.S. | https://www.embase.com/search/results?subaction=viewrecord&id=L2007107999&from=export U2 - L2007107999 | English |
| White Spot Lesions: A Serious but Often Ignored Complication of Orthodontic Treatment | 2022 | Open Dentistry Journal | Marya, A. and Venugopal, A. and Karobari, M.I. and Rokaya, D. | https://www.embase.com/search/results?subaction=viewrecord&id=L2016985288&from=export U2 - L2016985288 | English |
| Autism and Paediatric Dentistry: A Scoping Review. | 2019 | Oral health & preventive dentistry | Herrera-Moncada M and Campos-Lara P and HernÃ¡ndez-Cabanillas JC and Bermeo-Escalona JR and Pozos-GuillÃ©n A and Pozos-GuillÃ©n F and Garrocho-Rangel JA | https://pubmed.ncbi.nlm.nih.gov/31209442/ | English |
| A Prospective Cohort Study Assessing the Impact of Fixed Orthodontic Appliances on Saliva Properties and Oral Microbial Flora. | 2021 | Oral health & preventive dentistry | Kouvelis G and Papadimitriou A and Merakou K and Doulis I and Karapsias S and Kloukos D | https://pubmed.ncbi.nlm.nih.gov/33491380/ | English |
| Oral health status of children aged 6-12 years from the Danube Delta Biosphere Reserve. | 2012 | Oral health and dental management | Jipa IT and Amariei CI | https://pubmed.ncbi.nlm.nih.gov/22488031/ | English |
| Effectiveness of a fluoride-releasing orthodontic primer in reducing demineralization around bracketsâ€“a systematic review | 2021 | Orthodontic Waves | Maliael, M.T. and Subramanian, A.K. and Srirengalakshmi, M. | https://www.scopus.com/inward/record.uri?eid=2-s2.0-85121380913&doi=10.1080%2f13440241.2021.2007678&partnerID=40&md5=505af1f212ef82172546cf024ee3b005 | English |
| The effect of antimicrobial agents on bond strength of orthodontic adhesives: a meta-analysis of in vitro studies. | 2016 | Orthodontics & Craniofacial Research | Altmann AS and Collares FM and Leitune VC and Samuel SM | https://pubmed.ncbi.nlm.nih.gov/26257400/ | English |
| Effects of interproximal enamel reduction techniques used for orthodontics: A systematic review. | 2022 | Orthodontics & Craniofacial Research | GÃ³mez-Aguirre JN and Argueta-Figueroa L and Castro-GutiÃ©rrez MEM and Torres-Rosas R | https://pubmed.ncbi.nlm.nih.gov/34865310/ | English |
| Prevention of orthodontic enamel demineralization: A systematic review with meta-analyses | 2019 | Orthodontics & Craniofacial Research | Tasios, T. and Papageorgiou, S.N. and Papadopoulos, M.A. and Tsapas, A. and Haidich, A.-B. | https://www.embase.com/search/results?subaction=viewrecord&id=L627824220&from=export U2 - L627824220 | English |
| Significance of assessing risk factors for caries in their prevention. | 2002 | Orvosi hetilap | GÃ¡bris K and NyÃ¡rasdy I and BÃ¡nÃ³czy J | https://pubmed.ncbi.nlm.nih.gov/12138644/ | Hungarian |
| Oral health status of students at the age of 12-15 years in southern Highlands Province of Papua New Guinea: results of a descriptive epidemiological study. | 1997 | Papua and New Guinea medical journal | Bandara KM | https://pubmed.ncbi.nlm.nih.gov/10750412/ | English |
| Comparison of gingival and dental indices in lactating and non- lactating mothers during first 6 month after delivery | 2019 | Pesquisa Brasileira em Odontopediatria e Clínica Integrada | Aghazadeh, Z. and Behroozian, A. and Najafi, H. and Faramarzi, M. | https://www.embase.com/search/results?subaction=viewrecord&id=L2002554962&from=export U2 - L2002554962 | English |
| Inclusion of initial caries lesions in a population-based sample of Brazilian preschool children: Impact on estimates and treatment needs | 2020 | PLoS ONE | De Carvalho, P. and BÃ¶necker, M. and Tello, G. and Abanto, J. and Oliveira, L.B. and Braga, M.M. | https://www.embase.com/search/results?subaction=viewrecord&id=L2006825369&from=export U2 - L2006825369 | English |
| Fluoride release from two types of fluoride-containing orthodontic adhesives: Conventional versus resin-modified glass ionomer cements-An in vitro study. | 2021 | PLoS ONE | Dziuk Y and Chhatwani S and MÃ¶hlhenrich SC and Tulka S and Naumova EA and Danesh G | https://pubmed.ncbi.nlm.nih.gov/33635885/ | English |
| National strategy for prevention of oral diseases in children from 0 to 14 years old age in the Republic of Macedonia for the period 2008-2018. | 2013 | Prilozi | Sarakinova O and Carcev M and Getova B and Carceva Salja S and Janevska S | https://pubmed.ncbi.nlm.nih.gov/24280788/ | English |
| Long-term clinical and bacterial effects of xylitol on patients with fixed orthodontic appliances. | 2015 | Progress in Orthodontics | Masoud MI and Allarakia R and Alamoudi NM and Nalliah R and Allareddy V | https://pubmed.ncbi.nlm.nih.gov/26467791/ | English |
| Clinical evaluation of marketed orthodontic products: are researchers behind the times? A meta-epidemiological study | 2017 | Progress in Orthodontics | Seehra, J. and Pandis, N. and Fleming, P.S. | https://www.embase.com/search/results?subaction=viewrecord&id=L616505011&from=export U2 - L616505011 | English |
| Effects of various remineralizing agents on the outcome of post-orthodontic white spot lesions (WSLs): a clinical trial. | 2016 | Progress in Orthodontics | Singh S and Singh SP and Goyal A and Utreja AK and Jena AK | https://pubmed.ncbi.nlm.nih.gov/27480987/ | English |
| The influence of different types of brackets and efficacy of two chlorhexidine mouthwashes on oral hygiene and the incidence of white spot lesions in adolescents during the orthodontic therapy | 2016 | Psychiatria Danubina | Jurisic S, Kozomara D, Juric H, Verzak Z & Jurisic G | <https://www.embase.com/search/results?subaction=viewrecord&id=L614705521&from=export> | English |
| Diagnostic accuracy of parents' ratings of their child's oral health-related quality of life. | 2017 | Quality of life research | Reissmann DR and John MT and Sagheri D and Sierwald I | https://pubmed.ncbi.nlm.nih.gov/27743331/ | English |
| Incidence of caries in various forms fo dysgnathia. (3. Users of orthodontic appliances). | 1967 | Rassegna internazionale di stomatologia pratica | Crosetto E and Lukacs A | https://pubmed.ncbi.nlm.nih.gov/4386681/ | Italian |
| Demineralisation around orthodontic brackets- A review | 2016 | Research Journal of Pharmacy and Technology | Aravind, N. and Pandiyan, S. | https://www.embase.com/search/results?subaction=viewrecord&id=L619128533&from=export U2 - L619128533 | English |
| Oral health management in dental specialty clinics in Ecuador | 2020 | Revista Venezolana de Gerencia | Sofia, S.R. and Nelly Patricia, A.R. | <https://www.scopus.com/inward/record.uri?eid=2-s2.0-85089504204&doi=10.37960%2frvg.v25i3.33362&partnerID=40&md5=8e7a8d1dbff792dd9aef421319fcf61a> | Spanish |
| Caries associated microflora in plaque from orthodontic appliances retained with glass ionomer cement. | 1992 | Scandinavian journal of dental research | Hallgren A and Oliveby A and Twetman S | https://pubmed.ncbi.nlm.nih.gov/1631481/ | English |
| Dental findings in patients with Aarskog syndrome. | 1979 | Scandinavian journal of dental research | Halse A and Bjorvatn K and Aarskog D | https://pubmed.ncbi.nlm.nih.gov/295484/ | English |
| Caries incidence and orthodontic treatment with fixed appliances. | 1971 | Scandinavian journal of dental research | Zachrisson BU and Zachrisson S | https://pubmed.ncbi.nlm.nih.gov/5285724/ | English |
| A novel method quantifying caries following orthodontic treatment. | 2021 | Scientific reports | Erbe C and Hartmann L and Schmidtmann I and Ohlendorf D and Wehrbein H | <https://pubmed.ncbi.nlm.nih.gov/34725354/> | English |
| Fluoride varnish, ozone and octenidine reduce the incidence of white spot lesions and caries during orthodontic treatment: randomized controlled trial | 2022 | Scientific Reports | Katarzyna Grocholewicz, Paulina Mikłasz, Alicja Zawiślak, Ewa Sobolewska, Joanna Janiszewska‑Olszowska | <https://www.scopus.com/inward/record.uri?eid=2-s2.0-85136053828&doi=10.1038%2fs41598-022-18107-w&partnerID=40&md5=0515d8103012ddc8c1e7fdc574b757f2> | English |
| A New Technique With Sodium Hypochlorite to Increase Bracket Shear Bond Strength of Fluoride-releasing Resin-modified Glass Ionomer Cements: Comparing Shear Bond Strength of Two Adhesive Systems With Enamel Surface Deproteinization Before Etching | 2010 | Seminars in Orthodontics | Justus, R. and Cubero, T. and Ondarza, R. and Morales, F. | https://www.scopus.com/inward/record.uri?eid=2-s2.0-77649328922&doi=10.1053%2fj.sodo.2009.12.006&partnerID=40&md5=7725e64af2f05618f09683c4e0226d55 | English |
| White Spot Lesions After Orthodontic Treatment | 2008 | Seminars in Orthodontics | Willmot, D. | https://www.scopus.com/inward/record.uri?eid=2-s2.0-49849100824&doi=10.1053%2fj.sodo.2008.03.006&partnerID=40&md5=9c18b5158cbfb7ec0843a20e6a667386 | English |
| Incidence and risk factors of temporomandibular joint disorders in patients with bimaxillary protrusion after orthodontic treatment. | 2021 | Shanghai journal of stomatology | Cheng J and Zhang D and Xie LL and Wang P and Li J and Hao W | https://pubmed.ncbi.nlm.nih.gov/34693442/ | chi |
| The application of air abrasion in dentistry. | 2014 | Srpski arhiv za celokupno lekarstvo | MandiniÄ‡ Z and VuliÄ‡eviÄ‡ ZR and Beloica M and RadoviÄ‡ I and MandiÄ‡ J and CareviÄ‡ M and TekiÄ‡ J | https://pubmed.ncbi.nlm.nih.gov/24684041/ | Spanish |
| The extraction of the 6-year molars in orthodontic practice. | 1978 | Stomatologie der DDR | Henkert D | https://pubmed.ncbi.nlm.nih.gov/274840/ | German |
| Incidence of caries and periodontal condition in patients with cleft lip and cleft palate. | 1979 | Stomatologie der DDR | Huth A and Richter W | https://pubmed.ncbi.nlm.nih.gov/298403/ | German |
| Relationships between occlusal anomalies and caries, periodontal diseases, oral hygiene and temporomandibular joint disorders in 401 dental students (Berlin 1974). | 1978 | Stomatologie der DDR | Richter W | https://pubmed.ncbi.nlm.nih.gov/274846/ | German |
| The effectiveness of dentoalveolar anomalies treatment in children during early mixed dentition period. | 2019 | Stomatologiia | Olesov EE and Kaganova OS and Novozemceva TN and Shmatov KV and Olesova VN and Ivanov AS | https://pubmed.ncbi.nlm.nih.gov/31922513/ | Russian |
| The prevention of dental caries during adolescent orthodontic treatment. | 1989 | Stomatologiia | VodolatskiÄ­ MP and Khristoforando IuD | https://pubmed.ncbi.nlm.nih.gov/2533750/ | Russian |
| Incidence of dental caries, dental calculus, gingivitis, and orthodontic abnormalities in school children. | 1952 | Stomatologiia. Stomatology | IVANOV SB | https://pubmed.ncbi.nlm.nih.gov/13048983/ | Bulgarian |
| Cariostatic effect of glass ionomer retained orthodontic appliances. An in vivo study. | 1997 | Swedish dental journal | Twetman S and McWilliam JS and Hallgren A and Oliveby A | <https://pubmed.ncbi.nlm.nih.gov/9472145/> | English |
| Dental care of autistic children within the non-specialized Public Dental Service. | 2001 | Swedish dental journal | Fahlvik-Planefeldt C and HerrstrÃ¶m P | https://pubmed.ncbi.nlm.nih.gov/11813447/ | English |
| The effect of a low fluoride containing toothpaste on the development of dental caries and microbial composition using a caries generating model device in vivo. | 1995 | Swedish dental journal | Petersson, L.G. and Edwardsson, S. and Koch, G. and Kurol, J. and Lodding, A. | https://www.scopus.com/inward/record.uri?eid=2-s2.0-0029201119&partnerID=40&md5=2246df95ce5ef38113e792f5cd02addd | English |
| Dental care behaviour in Switzerland. | 2019 | Swiss dental journal | Schneider C and Zemp E and Zitzmann NU | https://pubmed.ncbi.nlm.nih.gov/31032607/ | English |
| Effectiveness of remineralising agents in prevention and treatment of orthodontically induced white spot lesions: a protocol for a systematic review incorporating network meta-analysis. | 2019 | Systematic reviews | Hu H and Feng C and Jiang Z and Wang L and Shrestha S and Su X and Shu Y and Ge L and Lai W and Hua F and Long H | https://pubmed.ncbi.nlm.nih.gov/31884975/ | English |
| White defects on enamel: Diagnosis and anatomopathology: Two essential factors for proper treatment (part 1) | 2013 | Taches blanches de l'email : diagnostic et anatomopathologie : deux donnees indispensables pour bien les traiter | Denis, M. and Atlan, A. and Vennat, E. and Tirlet, G. and Attal, J.-P. | https://www.embase.com/search/results?subaction=viewrecord&id=L369445185&from=export U2 - L369445185 | English |
| Evaluation of tooth demineralization using laser-fluorescence in dental school patients undergoing orthodontic treatment: A clinical study. | 2022 | Technology and health care | Cerón-Zamora E, Scougall-Vilchis RJ, Lara-Carrillo E, Contreras-Bulnes R, Robles-Bermeo NL, Mendoza-Rodríguez M, Medina-Solís CE, Maupomé G. | <https://pubmed.ncbi.nlm.nih.gov/35661038/> | English |
| Orthodontic treatment, dental health, and oral health behavior in young Norwegian adults. | 1988 | The Angle orthodontist | Lervik T and Haugejorden O | <https://pubmed.ncbi.nlm.nih.gov/3207215/> | English |
| The efficacy of fluoride varnish vs a filled resin sealant for preventing white spot lesions during orthodontic treatment. | 2022 | The Angle orthodontist | Flynn LN and Julien K and Noureldin A and Buschang PH | <https://pubmed.ncbi.nlm.nih.gov/34679162/> | English |
| Orthodontic appliances did not increase risk of dental caries and periodontal disease under preventive protocol. | 2019 | The Angle orthodontist | Bergamo AZN and de Oliveira KMH and Matsumoto MAN and Nascimento CD and Romano FL and da Silva RAB and da Silva LAB and Nelson-Filho P | https://pubmed.ncbi.nlm.nih.gov/30239218/ | English |
| Impact of malocclusion and dentofacial anomalies on the prevalence and severity of dental caries among adolescents. | 2015 | The Angle orthodontist | Feldens CA and Dos Santos Dullius AI and Kramer PF and Scapini A and Busato AL and Vargas-Ferreira F | https://pubmed.ncbi.nlm.nih.gov/26516712/ | English |
| Usefulness of an app in improving oral hygiene compliance in adolescent orthodontic patients. | 2016 | The Angle orthodontist | Zotti F and Dalessandri D and Salgarello S and Piancino M and Bonetti S and Visconti L and Paganelli C | https://pubmed.ncbi.nlm.nih.gov/25799001/ | English |
| Nanoparticles! armour against white spot lesions | 2021 | The Internal Medicine Journal | Aileni, K.R. and Shubha, A.B. and Patil, S.R. and Tabassum, S. and Bandela, V. and Alam, M.K. | https://www.embase.com/search/results?subaction=viewrecord&id=L2014310942&from=export U2 - L2014310942 | English |
| Drooling of saliva and its effect on the oral health status of children with cerebral palsy. | 2008 | The Journal of clinical pediatric dentistry | Hegde AM and Shetty YR and Pani SC | https://pubmed.ncbi.nlm.nih.gov/18524275/ | English |
| A Survey of Pediatric Dentists on the Treatment Timing and Modalities for White Spot Lesions in the United States. | 2019 | The Journal of clinical pediatric dentistry | Saito T and Park JH and Bay C | https://pubmed.ncbi.nlm.nih.gov/30520700/ | English |
| Dental Caries and Risk Factors in Swedish Adolescents about to Start Orthodontic Treatment with Fixed Appliances. | 2019 | The journal of contemporary dental practice | Baeshen HA and Rangmar S and Kjellberg H and Birkhed D | https://pubmed.ncbi.nlm.nih.gov/31316013/ | English |
| Sealants and White Spot Lesions in Orthodontics: A Review. | 2020 | The journal of contemporary dental practice | Linjawi AI | https://pubmed.ncbi.nlm.nih.gov/33020368/ | English |
| Caries management by risk assessment in a cleft and craniofacial center. | 2014 | The Journal of craniofacial surgery | Gaudilliere D and Thakur Y and Ku M and Kaur A and Shrestha P and Girod SC | https://pubmed.ncbi.nlm.nih.gov/25377980/ | English |
| Preventive and interceptive orthodontic needs among Syrian children. | 2016 | The Journal of the Egyptian Public Health Association | Burhan AS and Nawaya FR | https://pubmed.ncbi.nlm.nih.gov/27455087/ | English |
| Factors related to tooth loss among industrial workers in Phathum Thani, Thailand. | 2017 | The Southeast Asian journal of tropical medicine and public health | Jaaidee J and Chatrchaiwiwatana S and Ratanasiri A | https://pubmed.ncbi.nlm.nih.gov/29645412/ | English |

**Table S4.** Studies excluded after full text screening.

| **Title** | **Year** | **Journal** | **Authors** | **URL** | **Language** | **Reason of exclusion** |
| --- | --- | --- | --- | --- | --- | --- |
| A comparison of white spot lesion formation between a self-ligating bracket and a conventional preadjusted straight wire bracket. | 2008 | World Journal of Orthopedics | Polat O., Gokcelik A., Arman A., Arhun N. | <https://www.embase.com/search/results?subaction=viewrecord&id=L355915849&from=export> | English | Wrong data presentation |
| Association between fixed orthodontic treatment and dental caries: a 1-year longitudinal study. | 2020 | Brazilian oral research | Pinto AS and Alves LS and Maltz M and Zenkner JEDA | <https://pubmed.ncbi.nlm.nih.gov/33206775/> | English | Wrong study design |
| Clinical and salivary findings in patients with different types of orthodontic brackets | 2019 | Acta Stomatologica Croatica | Jurela, A. and SudareviÄ‡, K. and Budimir, A. and Brailo, V. and Brzak, B.L. and JankoviÄ‡, B. | <https://www.scopus.com/inward/record.uri?eid=2-s2.0-85072542687&doi=10.15644%2fasc53%2f3%2f4&partnerID=40&md5=5136886bd8753df2fdca3109b497bee4> | English | Wrong study design |
| Fluoride varnish for white spot lesion prevention during orthodontic treatment: results of a randomized controlled trial 1 year after debonding. | 2021 | European journal of orthodontics | Sonesson M and Brechter A and Lindman R and Abdulraheem S and Twetman S | <https://pubmed.ncbi.nlm.nih.gov/33009565/> | English | Wrong study design |
| Incidence of white spot lesions among patients treated with self- and conventional ligation systems. | 2015 | Clinical oral investigations | Akin M and Tezcan M and Ileri Z and Ayhan F | <https://pubmed.ncbi.nlm.nih.gov/25502686/> | English | Wrong study design |
| A practice-based evaluation of the prevalence and predisposing etiology of white spot lesions | 2016 | Angle Orthodontist | Brown, M.D. and Campbell, P.M. and Schneiderman, E.D. and Buschang, P.H. | <https://www.embase.com/search/results?subaction=viewrecord&id=L618361881&from=export> | English | Wrong type of study |
| Clinical efficacy of fluoride-releasing dental adhesive on restricting white spot lesion: An in vivo study | 2020 | International Journal of Research in Pharmaceutical Sciences (IJRPS) | Doss, C.A.V. and Tandon, A. and Sadath, S.M. and Palanivel, R.M. and Sakthivel, M. and Awan, M.K.S. | <https://www.embase.com/search/results?subaction=viewrecord&id=L2004813088&from=export> | English | Wrong type of study |
| Effect of calcium fluoride nanoparticles in prevention of demineralization during orthodontic fixed appliance treatment: a randomized clinical trial | 2022 | European journal of orthodontics | Al Tuma, R.R. and Yassir, Y.A. | <https://www.embase.com/search/results?subaction=viewrecord&id=L638911996&from=export> | English | Wrong type of study |
| Effectiveness of a sealant compared with no sealant in preventing enamel demineralization in patients with fixed orthodontic appliances: A prospective clinical trial | 2013 | American journal of orthodontics and dentofacial orthopedics | O'Reilly, M.T. and De JesÃºs ViÃ±as, J. and Hatch, J.P. | <https://www.embase.com/search/results?subaction=viewrecord&id=L369037151&from=export> | English | Wrong type of study |
| Enamel Demineralization during Fixed Orthodontic Treatment - Incidence and Correlation to Various Oral-hygiene Parameters. | 2007 | Journal of orofacial orthopedics | Lovrov S and Hertrich K and Hirschfelder U | <https://pubmed.ncbi.nlm.nih.gov/17882363/> | English | Wrong type of study |
| Factors Affecting the Formation, Severity and Location of White Spot Lesions during Orthodontic Treatment with Fixed Appliances. | 2014 | Journal of oral & maxillofacial research | Khalaf K | <https://pubmed.ncbi.nlm.nih.gov/24800054/> | English | Wrong type of study |
| Incidence of white spot lesions among patients treated with clear aligners and traditional braces | 2019 | Angle Orthodontist | Buschang, P.H. and Chastain, D. and Keylor, C.L. and Crosby, D. and Julien, K.C. | <https://www.embase.com/search/results?subaction=viewrecord&id=L627550588&from=export> | English | Wrong type of study |
| Lingual appliances reduce the incidence of white spot lesions during orthodontic multibracket treatment. | 2015 | American journal of orthodontics and dentofacial orthopedics | Dirk Wiechmann , Elisabeth Klang , Hans-Joachim Helms , Michael Knösel | <https://pubmed.ncbi.nlm.nih.gov/26321339/> | English | Wrong type of study |
| Occurrence and severity of enamel decalcification adjacent to bracket bases and sub-bracket lesions during orthodontic treatment with two different lingual appliances | 2016 | European journal of orthodontics | KnÃ¶sel, M. and Klang, E. and Helms, H.-J. and Wiechmann, D. | <https://www.embase.com/search/results?subaction=viewrecord&id=L619871961&from=export> | English | Wrong type of study |
| Orthodontics and enamel demineralization: clinical study of risk factors | 2014 | International Orthodontics | Benkaddour, A. and Bahije, L. and Bahoum, A. and Zaoui, F. | <https://www.embase.com/search/results?subaction=viewrecord&id=L609844310&from=export> | English | Wrong type of study |
| Prevalence of white spot lesion formation during orthodontic treatment | 2013 | Angle Orthodontist | Julien, K.C. and Buschang, P.H. and Campbell, P.M. | <https://www.embase.com/search/results?subaction=viewrecord&id=L369483929&from=export> | English | Wrong type of study |
| Risk factors for incidence and severity of white spot lesions during treatment with fixed orthodontic appliances | 2010 | American journal of orthodontics and dentofacial orthopedics | Chapman, J.A. and Roberts, W.E. and Eckert, G.J. and Kula, K.S. and GonzÃ¡lez-Cabezas, C. | <https://www.embase.com/search/results?subaction=viewrecord&id=L359336058&from=export> | English | Wrong type of study |
| Prevalence of white spot lesions and gingival index during orthodontic treatment in an academic setting | 2023 | American journal of orthodontics and dentofacial orthopedics | Sharab L, Loss C, Jensen D, Kluemper GT, Alotaibi M, Nagaoka H. | https://pubmed.ncbi.nlm.nih.gov/36720655/ | English | Wrong study design |

**Table S5.** Extended version of Table 2

| **Author (Year)** | **Age**  **m±SD (Range)** | **M/F** | **N subject evaluated (total study sample)** | **N teeth evaluated (total study sample)** | **Type of teeth** | **Study design** | **Baseline ICLs** | **Modality of ICLs assessment** | **ICLs index** | **Drying before evaluation** | **Other evaluation at baseline** | **Fluoride supplement evaluation** | **water fluoridation evaluation** | **Prevalence/ Incidence** | **Primary outcome** | **Secondary outcome** | **Results of primary outcome** | **Results of secondary outcome** |
| --- | --- | --- | --- | --- | --- | --- | --- | --- | --- | --- | --- | --- | --- | --- | --- | --- | --- | --- |
| Toti et al. (2022) | 17.4±5.1 (11-33) | 29/45 | 74 | 1776 | 16-46 | before-after treatment | n.a. | visual | ICDAS | yes | oral hygiene habits | no | no | prevalence | ICLs prevalence and distribution | correlation between ICLs and tooth brushing habits | prevalence 60.8%; surface prevalence 9.96%; mean 2.39±2.97 | higher frequency of daily toothbrushing was associated to a lower number of ICLs |
| Pinto et al. (2018) | n.a.  (10-30) | 78/117 | 195 (260) | n.a. | 17-47 | no treatment *vs* 1year *vs* 2 years vs, 3 years | n.a. | visual | Maltz | yes | oral hygiene, mother's education, family income | yes | yes | prevalence | ICLs prevalence | influence of treatment duration on ICLs | prevalence 1 year: 27.7%; mean 0.61I _95_CI[0.44;0.84]  2 years: 72.3%; mean 2.14 _95_CI[1.80;2.53]  3 years: 72.3; mean 1.95 _95_CI[1.63;2.32] | ICLs prevalence and caries activity is associated with treatment duration regardless of sociodemographic parameters and oral hygiene habits |
| Jiang et al. (2013) | 14.2±2.53 (10-20) | 23/24 | 47 (95) | 845 (1685) | 15-45 | placebo *vs* fluoride foam | yes | visual | Gorelick | yes | DMFT, DMFS | yes | yes | both | ICLs prevalence and incidence | ICLs distribution between groups | prevalence 64%; mean 4.79±5.58 – incidence 51%; mean 4.36±5.41 | acidulated phosphate fluoride foam is effective in reducing ICLs severity |
| Jiang et al. (2015) | 17.5±4.6 (11-24) | 60/142 | 202 | 5612 | 17-47 | ICLs evaluation at debonding | n.a. | visual | Gorelick | yes | oral hygiene, diet, socioeconomic status | yes | no | prevalence | ICLs prevalence | risk factor for ICLs | prevalence 57.9%; surface prevalence 17.3%; mean 4.8 | age, treatment duration, frequency of toothbrushing change in unhealthy dietary habits and consumption of sugary food are associated with higher prevalence |
| Martignon et al. (2010) | 18±5  (12-29) | 34/40 | 74 (137) | n.a. | 17-47 | treatment vs no treatment | n.a. | visual | ICDAS | yes | oral hygiene, DMFS | no | no | prevalence | ICLs prevalence | plaque and horal hygiene habits, DMFS | prevalence 96%; mean 11.3±7.1; | worse oral hygiene in group under orthodontic treatment; DMFS 6.7±6.3 |
| Hammad and Knösel (2016)* | 14.66 ±1.90  (12-18) | 12/9 | 21 (42) | n.a. | 15-45 | control *vs* sealant | no | photo | non-standardized | n.a. | oral hygiene | yes | no | incidence | ICLs incidence | oral hygiene | n.a. | oral hygiene is more effective in preventing ICLs than sealant |
| Heinig and Hartmann (2008) | 15.9  (11.6-39.5) | n.a. | 40 (78) | 800 (5788) | 15-45 | control vs sealant | yes | visual and photo | combined index system | yes | n.a. | yes | no | Incidence | ICLs incidence | ICLs distribution/severity; incidence of bracket loss | incidence 85.71%; surface incidence 9.18% | 55% moderate to severe lesion; higher incidence of brackets loss in control group |
| Gizani et al. (2016) | 15.9±3.9 (n.a.) | 33/10 | 43 (85) | n.a. | 15-25 | placebo vs probiotic *Lactobacillus reuteri* | yes | visual and photo | Gorelick | yes | oral hygiene, mutans streptococci count (MS), lactobacilli count (LB) | no | no | incidence | ICLs incidence | salivary MS and LB count, oral hygiene, adverse effects, compliance | incidence 39.53%; mean 1.7±2.5 | lower level of LB in both group at T1 was found, difference of MS level between T0 and T1 was found; probiotic was not effective in reducing ICLs incidence; low oral hygiene in both group |
| Ravikiran et al. (2021) | 20.6±4.79 (14-28) | 13/12 | 25 (50) | 250 (500) | 15-25 | control *vs* fluoride rinse | yes | photo | ad-hoc software | n.a. | n.a. | yes | n.a. | both | efficacy of amine fluoride mouthwash on ICLs reduction | n.a. | prevalence 2.46±1.87  –  incidence 0.55±0.43 | n.a. |
| Najafi et al. (2022) | 15.4±3.7 (12-20) | 15/14 | 29 (115) | 290 (1200) | 17-47 | placebo *vs* 10% xylitol *vs* 20% xylitol *vs* 5% fluoride varnish | no | visual | LF pen and Gorelick | yes | n.a. | no | no | incidence | ICLs incidence with LF pen and visual evaluation | adverse effect and patient satisfactionsurface incidence | surface incidence 31.4%  -  LF value mean 4.30±1.59 | fluoride and xylitol varnishes are effective in preventing ICLs. |
| Mohammed et al (2021) | n.a.  (15-30) | 41/79 | 120 (170) | n.a. | 15-45 | no treatment *vs* 6 mo treatment, *vs* 12 mo treatment | n.a. | visual | Gorelick | yes | n.a. | no | no | prevalence | ICLs prevalence | influence of sex | prevalence 6 mo 38,33%  prevalence 12 mo 46.66% | no sex differences although higher number of ICLs was found in female |
| Lucchese and Gherlone (2013) | 6 mo 9±1.3  12 mo 10±1.4 (n.a.) | 59/64 | 123 (191) | n.a. | 16-46 | no treatment *vs* 6 mo treatment, *vs* 12 mo treatment | n.a. | visual | Gorelick | yes | n.a. | no | no | prevalence | ICLs prevalence | ICLs distribution and influence of sex | prevalence 6 mo 40,67%  prevalence 12 mo 43,75% | no sex differences; higher prevalence in mandibular first molar and maxillary lateral incisor |
| Boersma et al. (2005) | 19.5±9.6 (n.a.) | 29/33 | 62 | 1536 | 16-46 | visual evaluation *vs* QLF evaluation | n.a. | visual and QLF | yes/no | n.a. | oral hygiene, diet, socioeconomic status, saliva test | yes | no | prevalence | QLF VS visual examination | correlation with caries risk factors (Diet, LB and MS count) and duration of the treatment | prevalence 97%; surface prevalence 18,48%  -  higher number of ICLs with QLF examination | higher prevalence in molars and premolars, in subject with gingival bleeding; correlation between LB and ICLs; no correlation for MB, age treatment duration, socioeconomic status and dietary habits |
| van der Kaaij et al. (2015)* | 13.6 (11.7-16.5) | 21/24 | 45 (81) | n.a. | 15-45 | placebo *vs* fluoride rinse | no | QLF imaging, visual | ICDAS | yes | ICDAS DMFS, oral hygiene | yes | no | incidence | ICLs incidence | ICLs incidence with QLF | mean ∆F= 10.3%± 3.0%. | mean lesion area=1.3 mm, SD 1.6.  ICDAS median 0.05 |
| Almosa et al. (2014) | G 22.5  P 21.2 (n.a.) | G 19/26  P 14/30 | 89 | G 822  P 831 | 15-45 | governmental (G) patients *vs* private (P) patients | yes | visual | ICDAS and LF pen | yes | n.a. | no | no | prevalence | ICLs prevalence with LF pen and visual evaluation | correlation between ICDAS and LF pen | G group  prevalence 91.1%; surface prevalence 50.2%  P group  prevalence 56.8%%; surface prevalence 15.3% | Spearman correlation between ICDAS and LF pen 0.71 |
| Stecksén-Blicks et al. (2007) | 14,3±1,6 (12-15) | n.a. | 125 (257) | n.a. | 15-25 | placebo *vs*: fluoride varnish | yes | photo | Gorelick | yes | DMFS | no | yes | both | ICLs prevalence and incidence | DMFS, ICLs progression and distribution | prevalence 29,7% -  incidence 25,7% | mean progression score 2.6±2.8; higher number of ICLs in lateral incisor, central incisor and premolars; no association between caries experience at baseline and ICLs incidence |
| Sonesson et al. (2014) | 14.6±1.65 (11_16) | 67/125 | 192 (380) | n.a. | 15-25 | control *vs* high-fluoride toothpaste | yes | visual | Gorelick | yes | n.a, | no | yes | both | ICLs prevalence and incidence | ICLs severity and distribution | prevalence 45,3%; mean 1.2±1.8  -  incidence 26,6% | 2.3% moderate to severe lesions; higher number of ICLs in lateral incisor, canines and premolars |
| Sonesson et al. (2020) | 13.8±1.8 (12-18) | 70/96 | 73 (148) | 730 (1480) | 15-25 | placebo *vs* fluoride varnish | yes | photo | Gorelick | yes | n.a. | no | yes | both | ICLs prevalence and incidence | ICLs severity and patient satisfaction | prevalence 43.83%; surface prevalence 43,56%  -  incidence 37.87%; mean 1.9±2.5 | 26,03% moderate to severe lesions |
| Esenlik et al. (2016) | 17.09±1.1 (16-18) | n.a. | 20 (40) | 542 (1088) | 17-47 | control *vs* CPP-ACP paste | no | photo | Gorelick | n.a. | DMFT, DMFS, oral hygiene | no | no | incidence | ICLs incidence | DMFT, DMFS, oral hygiene and ICLs distribution | incidence 80.00%; surface incidence 15.31%; mean 4.1±4.0 | DMFT 4.5; DMFS 5.5; mean severity 1.49; severe lesion 4.8%; higher number of ICLs in maxillary incisor, mandibular canines and premolars |
| Mahmoudzadeh et al. (2019) | n.a.  (12-30) | n.a. | 47 (95) | 276 (554) | 13-23 | control *vs* laser Co2 | yes | photo | ad hoc software and enamel decalcification | yes | n.a. | no | no | both | ICLs prevalence and incidence | ICLs extent | surface prevalence 15.2%  -  surface incidence 8.7% | n.a. |
| Tufekci et al. (2011) | 6 mo 17.4±1.3  12 mo 17.5±1.4  (>12) | 38/34 | 72 (100) | n.a. | 15-25 | no treatment *vs* 6 mo treatment, *vs* 12 mo treatment | n.a. | visual | non-standardised | yes | no | yes | no | prevalence | ICLs prevalence | influence of sex | prevalence 6 mo 37.83%; mean 0.92±0.22  prevalence 12 mo 45.71%; mean 1.13±0.22 | ICLs prevalence higher in male |
| *studies excluded from metanalysis: M±SD=mean ± standard deviation; N=number; ICLs=Initial Caries Lesions; n.a.= not available; _95_CI = 95% Confidence Interval; ICDAS= International Caries Detection System; DMFT=Decayed Missed Filled Teeth; DMFS= Decayed Missed Filled Surface; MS=mutans streptococci count; LM=lactobacilli count; LF=Laser Fluorescence; CPP-ACP= casein phosphopeptide amorphous calcium phosphate | | | | | | | | | | | | | | | | | | |

**Table S6.**Prevalence of white spot lesions in subject during or after fixed orthodontic appliance

| **№ of studies** | **Certainty assessment** | | | | | | **Effect** | | **Certainty** |
| --- | --- | --- | --- | --- | --- | --- | --- | --- | --- |
|  | **Study design** | **Risk of bias** | **Inconsistency** | **Indirectness** | **Imprecision** | **Other considerations** | **№ of individuals** | **Rate/Mean (95% CI)** |  |
| Prevalence rate per subject | | | | | | | | | |
| 4 | randomised trials | serious^a^ | serious^b^ | serious^c^ | serious^d^ | none | 437 | event rate 45.00% [33.00;57.00] | ⨁◯◯◯ Very low |
| 6 | NRSIs | very serious^e^ | serious^b^ | serious^c^ | serious^d^ | none | 673 | event rate 58.00% [45.00;69.00] | ⨁◯◯◯ Very low |
| 3 | observational studies | not serious | serious^b^ | serious^c^ | serious^d^ | none | 338 | event rate 74.00% [52.00;88.00] | ⨁◯◯◯ Very low |
| Prevalence rate per surface | | | | | | | | | |
| 2 | randomised trials | not serious | very serious^b^ | serious^c^ | serious^d^ | none | 1006 | event rate 27.00% [8.00;61.00] | ⨁◯◯◯ Very low |
| 1 | NRSIs | very serious^f^ | very serious^g^ | serious^c^ | very serious^h^ | none | 1653 | event rate 30.00% [7.00;70.00] | ⨁◯◯◯ Very low |
| 3 | observational studies | not serious | serious^b^ | serious^c^ | not serious | none | 8924 | event rate 15.00% [11.00;20.00] | ⨁⨁◯◯ Low |
| Mean prevalence per subject | | | | | | | | | |
| 3 | randomised trials | very serious^i^ | serious^b^ | serious^c^ | serious^d^ | none | 264 | mean 2.62 [1.06;4.18] | ⨁◯◯◯ Very low |
| 2 | NRSIs | serious^j^ | very serious^b^ | serious^c^ | very serious^d^ | none | 146 | mean 2.17 [1.55;2.80] | ⨁◯◯◯ Very low |
| 1 | observational studies | not serious | very serious^g^ | serious^c^ | very serious^h^ | none | 74 | mean 2.39 [1.71;3.07] | ⨁◯◯◯ Very low |

#### Explanations

a. 3 low risk and 1 high risk studies according to RoB 2

b. high heterogeneity due to different sample size, publication year, study location

c. individual caries risk assessment not evaluated

d. Body of evidence includes studies with few participants and there are wide confidence intervals around the estimate of the effect

e. 5 moderate risk and 1 severe risk studies according to ROBINS 1

f. 1 severe risk study according to ROBINS 1

g. only 1 study

h. Body of evidence includes only one study

i. 1 low risk and 2 high risk studies according to RoB 2

j. 2 moderate risk according to ROBINS 1

**Table S7.** Incidence rate of white spot lesions in subject during or after fixed orthodontic appliance

| **№ of studies** | **Certainty assessment** | | | | | | **Effect** | | **Certainty** |
| --- | --- | --- | --- | --- | --- | --- | --- | --- | --- |
|  | **Study design** | **Risk of bias** | **Inconsistency** | **Indirectness** | **Imprecision** | **Other considerations** | **№ of individuals** | **Rate/Mean [95% CI]** |  |
| Incidence rate per subject | | | | | | | | | |
| 6 | randomised trials | serious^a^ | serious^b^ | serious^c^ | serious^d^ | none | 493 | event rate 40.00% [29.00;52.00] | ⨁◯◯◯ Very low |
| 1 | observational studies | serious^e^ | very serious^f^ | serious^c^ | very serious^g^ | none | 40 | event rate 86.00% [71.00;94.00] | ⨁◯◯◯ Very low |
| Incidence rate per surface | | | | | | | | | |
| 3 | randomised trials | not serious | very serious^b^ | serious^c^ | serious^h^ | none | 1090 | event rate 17.00% [8.00;32.00] | ⨁◯◯◯ Very low |
| 1 | observational studies | serious^e^ | very serious^f^ | serious^c^ | very serious^g^ | none | 800 | event rate 9.00% [7.00;11.00] | ⨁◯◯◯ Very low |
| Mean incidence per subject | | | | | | | | | |
| 5 | randomised trials | serious^i^ | very serious^b^ | serious^c^ | serious^d^ | none | 208 | mean 2.29 [1.12;3.46] | ⨁◯◯◯ Very low |

#### Explanations

a. 4 low risk, 1 high risk and 1 some concerns according to RoB 2

b. high heterogeneity due to different sample size, publication year, study location

c. individual caries risk assessment not evaluated

d. Body of evidence includes studies with few participants and there are wide confidence intervals around the estimate of the effect

e. some concerns study according to ROBINS-I

f. only 1 study

g. Body of evidence includes only one study with few participant

h. Wide confidence intervals around to estimate the effect

i. 3 low risk, 1 high risk and 1 some concerns studies according to RoB 2

**Figure S1**. Publication bias of the 6 outcomes evaluated in the metanalysis.


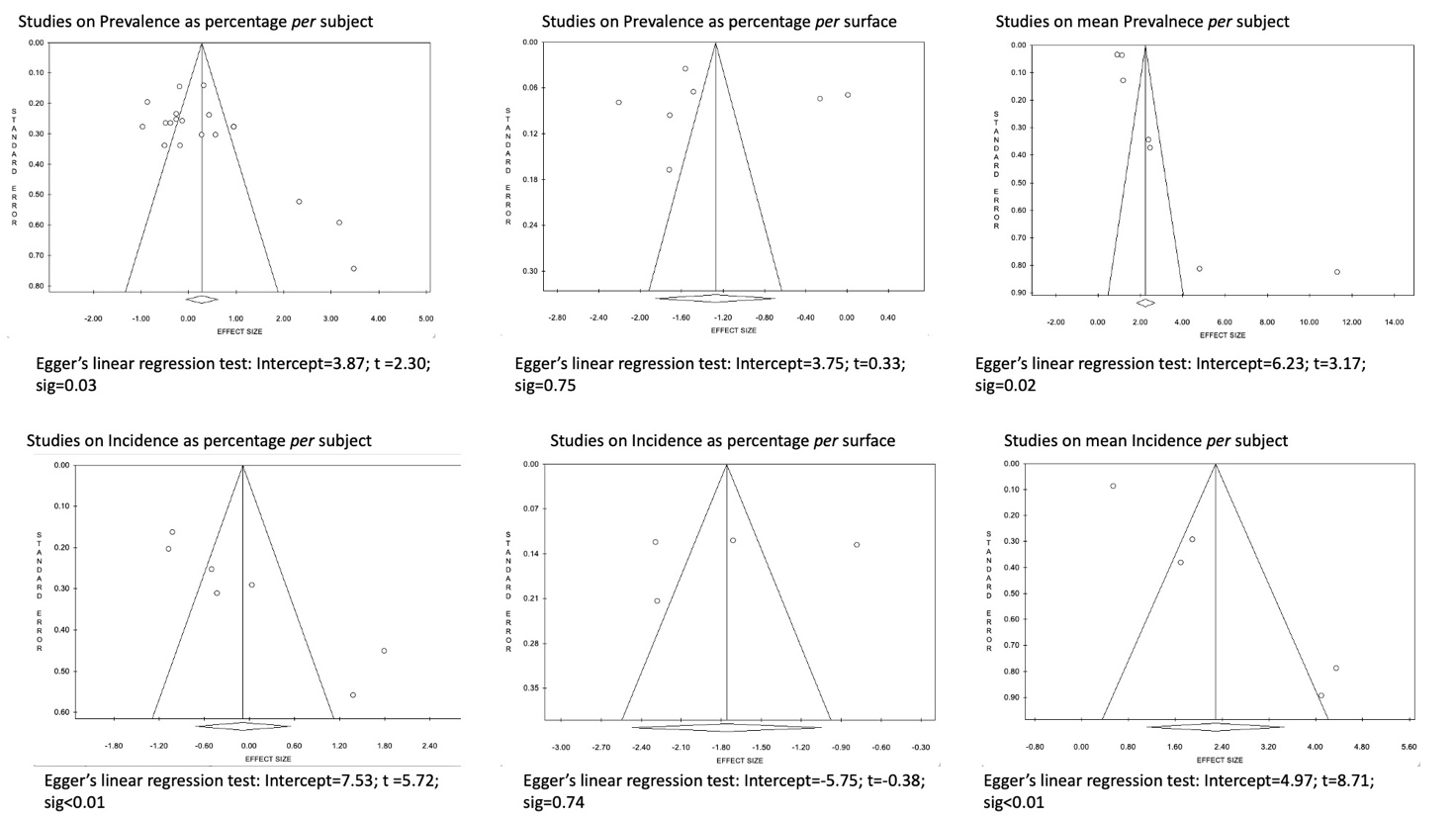

Supplement: cjae008_suppl_Supplementary_Material [file cjae008_suppl_supplementary_material.docx]
